# Hypertension and increased endothelial mechanical stretch promote monocyte differentiation and activation: roles of STAT3, interleukin 6 and hydrogen peroxide

**DOI:** 10.1093/cvr/cvy112

**Published:** 2018-05-23

**Authors:** Roxana Loperena, Justin P Van Beusecum, Hana A Itani, Noah Engel, Fanny Laroumanie, Liang Xiao, Fernando Elijovich, Cheryl L Laffer, Juan S Gnecco, Jonathan Noonan, Pasquale Maffia, Barbara Jasiewicz-Honkisz, Marta Czesnikiewicz-Guzik, Tomasz Mikolajczyk, Tomasz Sliwa, Sergey Dikalov, Cornelia M Weyand, Tomasz J Guzik, David G Harrison

**Affiliations:** 1 Department of Molecular Physiology and Biophysics, Vanderbilt University, Nashville, TN, USA; 2 Division of Clinical Pharmacology, Department of Medicine, Vanderbilt University Medical Center, Nashville, TN, USA; 3 Department of Biological Sciences, Vanderbilt University, Nashville, TN, USA; 4 Department of Pathology, Microbiology and Immunology, Vanderbilt University, Nashville, TN, USA; 5 Institute of Infection, Immunity & Inflammation, University of Glasgow, Glasgow, UK; 6 Institute of Cardiovascular and Medical Sciences, University of Glasgow, Glasgow, UK; 7 Department of Pharmacy, University of Naples Federico II, Naples, Italy; 8 Department of Internal Medicine, Jagiellonian University School of Medicine, Cracow, Poland; 9 Department of Immunology, Jagiellonian University School of Medicine, Cracow, Poland; 10 Division of Immunology and Rheumatology, Department of Medicine, Stanford University School of Medicine, Palo Alto, CA, USA

**Keywords:** STAT3, Intermediate monocyte, Endothelium, Interleukin 6, Nitric oxide

## Abstract

**Aims:**

Monocytes play an important role in hypertension. Circulating monocytes in humans exist as classical, intermediate, and non-classical forms. Monocyte differentiation can be influenced by the endothelium, which in turn is activated in hypertension by mechanical stretch. We sought to examine the role of increased endothelial stretch and hypertension on monocyte phenotype and function.

**Methods and results:**

Human monocytes were cultured with confluent human aortic endothelial cells undergoing either 5% or 10% cyclical stretch. We also characterized circulating monocytes in normotensive and hypertensive humans. In addition, we quantified accumulation of activated monocytes and monocyte-derived cells in aortas and kidneys of mice with Angiotensin II-induced hypertension. Increased endothelial stretch enhanced monocyte conversion to CD14^++^CD16^+^ intermediate monocytes and monocytes bearing the CD209 marker and markedly stimulated monocyte mRNA expression of interleukin (IL)-6, IL-1β, IL-23, chemokine (C-C motif) ligand 4, and tumour necrosis factor α. STAT3 in monocytes was activated by increased endothelial stretch. Inhibition of STAT3, neutralization of IL-6 and scavenging of hydrogen peroxide prevented formation of intermediate monocytes in response to increased endothelial stretch. We also found evidence that nitric oxide (NO) inhibits formation of intermediate monocytes and STAT3 activation. *In vivo* studies demonstrated that humans with hypertension have increased intermediate and non-classical monocytes and that intermediate monocytes demonstrate evidence of STAT3 activation. Mice with experimental hypertension exhibit increased aortic and renal infiltration of monocytes, dendritic cells, and macrophages with activated STAT3.

**Conclusions:**

These findings provide insight into how monocytes are activated by the vascular endothelium during hypertension. This is likely in part due to a loss of NO signalling and increased release of IL-6 and hydrogen peroxide by the dysfunctional endothelium and a parallel increase in STAT activation in adjacent monocytes. Interventions to enhance bioavailable NO, reduce IL-6 or hydrogen peroxide production or to inhibit STAT3 may have anti-inflammatory roles in hypertension and related conditions.

## 1. Introduction

In 2016, hypertension was ranked as the leading risk factor for global burden of disease in both developed and underdeveloped countries.^1^ In the past 10 years, it has become evident that activated immune cells infiltrate the kidney and other organs and that these cells contribute to the end-organ damage in this disease.^2^ In particular, monocytes seem to play a particularly important role in hypertension. Wenzel *et al.* showed selective ablation of lysozyme M-positive (LyzM^+^) myelomonocytic cells in mice completely prevented Angiotensin II (Ang II) induced hypertension and prevented the endothelial dysfunction and vascular oxidative stress generally observed in this model.^3^

The mechanism by which monocytes promote hypertension remains undefined but likely involves transformation into activated states or into other cell types, including macrophages and monocyte-derived dendritic cells (DCs). Indeed, De Ciuceis *et al.* found that mice lacking macrophage colony-stimulating factor, required for the stimulation of macrophage formation from monocytes, are protected against blood pressure (BP) elevation.^4^ Furthermore, these mice are protected from vascular remodelling, vascular superoxide production and the alteration of endothelium-dependent vasodilation that normally accompanies hypertension.^4^ Likewise, monocyte-derived DCs seem to play a critical role in hypertension. DCs potently activate T cells, which are essential for full development of hypertension.^5^ We have shown that in hypertension DCs accumulate isolevuglandin (IsoLG)-adducted proteins that are immunogenic, and that adoptive transfer of DCs from hypertensive mice primes hypertension in recipient mice. DCs of hypertensive mice produce large quantities of cytokines including IL-6, IL-23, and TNFα and exhibit enhanced ability to drive proliferation of T cells from other hypertensive mice.^6^ These cytokines are activated in response to the phosphorylation of signal transducer and activator of transcription 3 (STAT3),^7^ and their production can skew T cells towards T helper 17 (T_H_17) differentiation. The production of IL-17 by T cells is critical for maintenance of Ang II-induced hypertension and vascular dysfunction.^8^ Indeed, we have observed increased IL-17A producing T cells in the circulation of hypertensive humans.^9^

Circulating monocytes in humans have been classified into three subpopulations depending on their surface expression of the toll-like receptor 4 (TLR4) co-receptor CD14 and the FcγIII receptor CD16.^10^ Most circulating monocytes are classified as ‘classical’ and exhibit surface expression if CD14 and little or no CD16 (CD14^++^CD16^−^). These are thought to represent cells newly released from the bone marrow, and they circulate for approximately 1 day before either dying, transmigrating or transforming into another phenotype.^11^ Non-classical monocytes, characterized by their expression of CD16 and low levels of CD14 or CD14^low^CD16^++^, and are known to expand in inflammatory states. Upon stimulation, these CD14^low^CD16^++^ cells exhibit increased production of TNFα.^12^ In 1988, a small population of monocytes expressing both CD14 and CD16 was identified,^13^ subsequently termed intermediate monocytes or CD14^++^CD16^+^.^14^ These cells are also expanded in inflammatory states such as rheumatoid arthritis, psoriasis, and peripheral artery disease.^15–18^ Recent deuterium labelling studies indicate that intermediate and non-classical monocytes arise sequentially from the CD14 population.^11^ When placed in culture, classical monocytes acquire increasing levels of CD16 with time.^13^ The population of monocytes that gives rise to human monocyte-derived DCs remains poorly defined, but includes CD16^+^ cells.^19^ Another population are monocytes that express the DC-specific intracellular adhesion molecule (ICAM)-3 grabbing non-integrin (DC-SIGN) or CD209 DC receptor, which interacts with the leukocyte cell-derived chemotaxin 2 (LECT2). LECT2 is crucial for the process of adhesion and rolling of DCs on endothelial cells and can mediate macrophage activation and protection against bacterial sepsis.^20^ The expression of CD209 on DCs is considered a marker of maturation.^21^

A potential source of monocyte activation in hypertension is interaction of these cells with the vascular endothelium. In this regard, Randolph et al. showed that monocytes cultured with endothelial cells that had been stimulated with IL-1β, LPS, or zymosan particles differentiate into either DCs or macrophages depending on reverse transmigration through the endothelium.^22^ These investigators further showed that CD16^+^ cells are more likely to reverse transmigrate and that reverse transmigration of monocytes promotes the formation of a CD16^+^ population.^23^ A feature of hypertension that can activate the endothelium is increased mechanical stretch. Indeed, increased cyclic stretch activates transcription factors including AP1, the cAMP responsive binding protein, NFκB in human endothelial cells,^24^ and a variety of signalling molecules including ERK1/2, the focal adhesion kinase pp125fak, PI3 kinase, and p21Ras.^25–27^ Gene array studies have indicated that endothelial stretch increases expression of inflammatory mediators including IL-6, IL-8, the monocyte chemoattractant protein 1 (MCP-1) and the vascular cell adhesion molecule-1 (VCAM-1).^28^ These events promote monocyte adhesion, rolling, and transmigration through the endothelium,^29^ and some are redox sensitive.^30^ Hishikawa and Luscher showed that 10% stretch of human aortic endothelial cells (HAECs) enhances superoxide production, mediated initially by the NADPH oxidase and subsequently by the nitric oxide (NO) synthase depending on the presence of tetrahydrobiopterin.^31^

In the present study, we examined the role of endothelial mechanical stretch and STAT3 in promoting transformation of co-cultured human monocytes into the intermediate-phenotype and to cells bearing DC properties. We also examined monocyte subsets and STAT phosphorylation status in humans with hypertension. Our findings provide new insight into how altered mechanical forces in the vessel can promote immune activation.

## 2. Methods

### 2.1 Human subjects

We performed three studies: one involved obtaining monocytes from normotensive subjects to analyse their response to endothelial stretch. For this analysis, we included male and female normotensive participants who had BP between <135/80 mmHg. In a second study, we examined the phenotype of circulating monocytes from 20 normotensive subjects, 52 subjects with mild hypertension (systolic BP from 130 to 140 mmHg), and 60 subjects with more severe hypertension (systolic BP >140 mmHg). In a third study, we recruited 15 normotensive subjects and 12 hypertensive subjects for analysis of phospho-STAT levels in circulating monocytes. For this third study, participants were considered hypertensive if they had a systolic BP higher than 140 mmHg, a diastolic BP higher than 90 mmHg or had a diagnosis of hypertension and were currently treated with anti-hypertensive agents. Normal and hypertensive volunteers were included between ages 18 and 55 years.

Exclusion criteria included the following: (i) autoimmune disease or history of inflammatory diseases; (ii) recent vaccinations within the last 3 months; (iii) confirmed or suspected causes of secondary hypertension; (iv) severe psychiatric disorder; (v) HIV/AIDS; (vi) current treatment with steroids or antihistamines; (vii) liver or renal disease and (viii) history of cancer. Protocol 1 and 3 were approved by the Vanderbilt Institutional Review Board and conformed to standards of the US Federal Policy for the Protection of Human Subjects. The Ethics Committee of Jagiellonian University approved protocol two. Written informed consent was obtained from all patients.

### 2.2 Monocyte isolation and monocyte-HAECs cultures

HAECs (Lonza, Walkersville, MD, USA) were grown to confluency on flexible six-well culture plates that permit uniaxial stretch (Flexcell^®^ International Corporation, Burlington, NC, USA). These were coated with Collagen I and 1% gelatin crosslinked with 0.05% of glutaraldehyde. Cells were fed every other day with EBM-2 medium (Lonza) containing EGM-2 growth factors and supplements and 2% foetal bovine serum and stored in 37°C incubator with 5% CO_2_.

Peripheral blood mononuclear cells (PBMCs) from each volunteer were isolated initially by Ficoll-density gradient centrifugation and subsequently CD14^+^ monocytes were further isolated from PBMCs using negative selection with the monocyte isolation kit (Miltenyi Biotec 130-096-537; Miltenyi Biotec, Auburn, CA, USA) as previously described.^32^ Monocytes from each volunteer were added to the endothelial cells previously grown on Uniflex^®^ six-well culture plates so that we could simultaneously examine the response of monocytes to endothelial cells undergoing 5% and 10% uniaxial stretch. In indicated experiments, 6% and 8% stretch was applied. In some experiments, one million human monocytes were added to Uniflex^®^ six-well culture plates coated with Collagen I and Pronectin^®^ (RGD) (Flexcell^®^ International Corporation) in the absence of HAECs. Uniaxial cyclical mechanical stretch was applied to the endothelial cell monolayers at 5% and 10% elongation strain, 1 Hz, and ½ sine curve.

### 2.3 T-cell proliferation assay

One million CD14^+^ monocytes were exposed to endothelial cells undergoing either 5% or 10% stretch as described above. Forty-eight hours later, the endothelial cells were removed by Fluorescence Activated Cell-Sorting (FACS) using CD31 staining. The same subjects returned on this day, additional blood was sampled and T cells were harvested from PBMCs using negative selection (130-096-535; Miltenyi Biotec). These T cells were loaded with carboxyfluorescein succinimidyl ester (CFSE) (Invitrogen) and cultured with the sorted monocytes at a 1:10 ratio for 7 days. Proliferation was measured by the CFSE dilution using flow cytometry.

### 2.4 Animals

Wildtype (WT) C57Bl/6 male mice, obtained from The Jackson Laboratory were studied at 3 months of age. Ang II (490 ng/kg/min) or vehicle (sham) was infused for 6 days via osmotic minipumps (Alzet, Model 2002; DURECT Corporation, Cupertino, CA, USA) as previously described.^5^^,^^6^ All animal procedures were approved by Vanderbilt University’s Institutional Animal Care and Use Committee (IACUC) where the mice were housed and cared for in accordance with the Guide for the Care and Use of Laboratory Animals, US Department of Health and Human Services.

### 2.5 Flow cytometry

**Figure 1:**
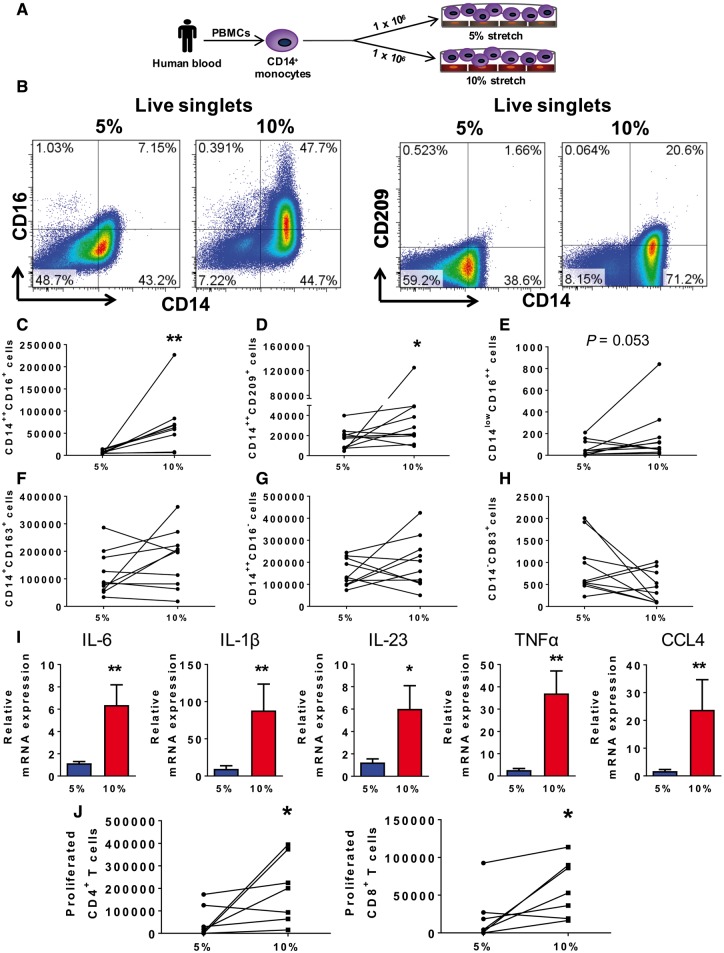
Hypertensive mechanical stretch in human endothelial cells promotes monocyte activation and differentiation. Human CD14^+^ monocytes were isolated by magnetic sorting from PBMCs of normal human volunteers and cultured with HAECs exposed to cyclical stretch. (*A*) Schematic of the experimental design and (*B*) gating strategy for phenotyping human monocytes including classical (CD14^++^CD16^−^), intermediate (CD14^++^CD16^+^) and non-classical monocytes (CD14^low^CD16^++^). (*C*) Changes in numbers of cells for each subject are depicted by connected lines for CD14^++^CD16^+^ (*n* = 9) and (*D*) CD14^++^CD209^+^, (*E*) CD14^low^CD16^++^, (*F*) macrophage population (CD14^+^CD163^+^), (*G*) CD14^++^CD16^−^, (*H*) CD14^−^CD83^+^ (*n* = 10). (*I*) Relative monocyte mRNA expression of IL-6, IL-1β, IL-23, TNFα, and CCL4 in adhered monocytes and in monocytes in suspension (5%, *n* = 15; 10%, *n* = 16). (*J*) Monocyte-HAECs cultures were stretched to either 5% or 10% for 48 h followed by sorting monocytes from HAECs using CD31^+^ isolation kit and FACS. Monocyte populations were cultured with CFSE-labelled T cells isolated from PBMCs of the same participants. Seven days later, we measured proliferation in the CD4^+^ and CD8^+^ T-cell populations by flow cytometry. Changes in number of proliferated CD4^+^ and CD8^+^ T cells after 7 days in culture for each subject are depicted by connected lines (*n* = 7). Comparisons were made using one-tail paired *t*-tests (*P <* *0.05, **P <* *0.01).

Monocyte populations were collected from the endothelial/monocyte co-cultures by digesting the Collagen I and 1% gelatin coating with Collagenase A and B (1 mg/mL) and DNAse I (100 μg/mL) in RPMI 1640 medium with 10% FBS for 30 min at 37°C. This procedure harvested not only cells in suspension, but also cells adhering to the endothelium and those that potentially transmigrated to the subendothelial collagen/gelatin layer. In mice, organs were transcardially perfused by physiological pressure with saline solution prior to organ collection. Single cell suspensions were prepared from aortas and kidneys as previously described.^33^ Flow cytometry was performed as previously described.^32^ Gates for each antibody stain were determined by flow minus one (FMO) controls and confirmed using isotype controls. We employed live/dead stains to eliminate non-viable cells and selected only single cells for analysis (*Figure 1A*). For the detection of murine monocytes, DCs and macrophages in the aorta and kidney we employed a gating strategy that effectively discriminates between these subsets as previously described.^34^ For freshly isolated human monocytes we used a gating strategy as described by Urbanski *et al.*^35^

### 2.6 Co-immunoprecipitation, quantitative RT-PCR and ELISA

For co-immunoprecipitation (co-IP) studies, monocytes were isolated from the monocyte-HAEC cultures using a negative selection for human CD31 (Miltenyi Biotec). co-IP was performed as previously described.^32^ The bands of interest were normalized to total STAT3. For RT-PCR monocytes were again isolated from the endothelium and lysed in RLT buffer and RNA was extracted using the RNeasy Mini Kit (Qiagen, Germantown, MD, USA) and RT-PCR was performed according to the manufacturer’s instructions using both iScript cDNA synthesis kit (BioRad, Hercules, CA, USA) or the High Capacity cDNA reverse transcriptase kit (Applied Biosystems, Foster City, CA, USA). Samples were evaluated for human IL-6, IL-1β, IL-23, TGF-β, CD168, p22^*phox*^, CCL4, IL-18, CCL2, MMP8, and TNFα using Taqman primers. Relative quantification was determined using the comparative CT method where samples were normalized to GAPDH and calibrated to the average of the control group (5% stretch). LECT2, IL-6, and High Mobility Group Box-1 (HMGB-1) protein were quantified using ELISA kits from LS-Bio, Affymetrix and IBL International, respectively.

### 2.7 Visualization of monocytes with endothelial cells in co-cultures

One to two million human monocytes isolated from PBMCs of normotensive people were incubated with 12 µM of CellTracker™ Green CMFDA Dye (C2925, ThermoFisher Scientific, Waltham, MA, USA) in EBM2 medium (Lonza) for 30 min at 37°C according to the manufacturer’s instructions. After incubation, cells were washed with medium and one million fluorescently tagged monocytes were added to wells containing either confluent HAECs on Collagen I/gelatin coated six-well stretch plates or without the presence of endothelial cells in six-well stretch plates coated with Collagen I or Pronectin^®^ (RGD, Flexcell^®^ International Corporation). For imaging, endothelial-containing cultures or monocyte alone cultures were exposed to either 5% or 10% levels of continuous uniaxial stretch for 24 h. Cells were subsequently washed with 1× PBS and the remaining cells were fixed with 4% paraformaldehyde (PFA) solution for 15 min at room temperature. The cells were then permeabilized with 0.5% Triton X-100 (Sigma) in 1× PBS for 30 min at room temperature. Cells underwent several washes in PBS and were blocked with goat serum solution (5% goat serum and 2.3% glycine in PBS) for 1 h at room temperature. Cultures with endothelial cells were then stained with the primary purified anti-human CD54 or ICAM-1 (HA58, Biolegend, San Diego, CA, USA) at a 1:100 concentration in goat serum solution for 1 h at room temperature. Cells were then stained with the ReadyProbes secondary antibodies conjugated with Alexa Fluor™ 594 dyes goat anti-mouse antibody (R37121, ThermoFisher Scientific) using one drop per millilitre of PBS and incubation for 30 min at room temperature. Finally, all plates’ flexible membranes were mounted unto glass slides and ProLong^®^ Gold antifade reagent with DAPI (ThermoFisher Scientific) was used to stain for the nucleus before adding the coverslip. Imaging of the slides was performed using an EVOS™ FL Imaging System (ThermoFisher Scientific). Adhered monocytes were counted using ImageJ software in three random fields and an average was calculated and used as a graphing representative.

For imaging with confocal microscopy membranes were fixed in 4% PFA for 15 min at room temperature, washed with PBS and then blocked for one hour in Dako Serum Free Protein Block (DSFPB) (Agilent Technologies, Santa Clara, CA). Membranes were incubated with purified anti-CD83 antibody (BioLegend; category number 305301) or isotype matched control (Biolegend; category number 400102) in DSFPB overnight at 4°C, washed in PBS, and then probed with Alexa Fluor 647 labelled anti-Murine IgG raised in goat (ThermoFisher Scientific) for 30 min. Following washing in PBS, membranes were stained with anti-CD31-FITC (BioLegend; category number 303103) and anti-CD14-Alexa Fluor 594 (BioLegend; category number 325630) for 2 h at room temperature. Samples were washed in PBS and counterstained with DAPI for 10 min. Membranes were washed in PBS and then mounted on glass-bottomed dishes. Immunofluorescent images were then acquired using a Zeiss Cell Observer SD confocal fluorescent microscope (Zeiss, Oberkochen, Germany).

### 2.8 Visualization of monocytes/macrophages in aortic sections of WT C57Bl/6 mice

C57Bl/6 mice were infused with either sham or Ang II for 2 weeks and aortas were harvested and embedded in paraffin for sectioning. Paraffin sections were dehydrated with ethanol and fixed with 10% formalin for 20 min at room temperature. The cells were then permeabilized with 0.5% Triton X-100 (Sigma) in 1X PBS for 30 min at room temperature. Cells underwent several washes in PBS and were blocked with foetal serum solution (5% goat serum and 2.3% glycine in PBS) for 1 h at room temperature. Sections were stained with the primary purified anti-mouse F4/80 and pSTAT3 (Y705) at a 1:100 and 1:50, respectively, in serum at 4°C overnight. Cells were then stained with secondary antibodies conjugated Alexa Fluor™ donkey anti-rat and anti-rabbit antibodies (Invitrogen) using one drop per millilitre of PBS and incubation for 1 h and 30 min at room temperature. Finally, we added ProLong^®^ Gold antifade reagent with DAPI (ThermoFisher Scientific) to stain for the nucleus before adding the coverslip. Imaging of the slides was performed using an EVOS™ FL Imaging System (ThermoFisher Scientific).

### 2.9 Reagents

All reagents were obtained in the highest purity available. These included Stattic, Tempol, Tofacitinib citrate, PEG-Catalase, Angiotensin II and Ebselen from Sigma, DETA-NONOate from Cayman Chemical Company, and the anti-IL-6 neutralizing antibody was from BioLegend (clone MQ2-13A5). l-Nitroarginine methyl ester (l-NAME) was from Abcam.

### 2.10 Statistics

All data are expressed as mean ± SEM. One tailed-paired and unpaired Student’s *t*-tests were used to compare two groups. In case of the non-normality, the non-parametric test one-tailed Mann–Whitney *U* test was used. When examining the effect of varying endothelial percent stretch on monocyte transformation to the intermediate phenotype, we employed one-way ANOVA with Student Newman Keuls post hoc test. When examining the effect of DETA-NONOate, we employed the Friedman’s multiple comparison test followed by the Dunn’s post hoc test. To compare the distribution of monocyte subtypes in normotensive humans vs. humans with mild or severe hypertension, we employed one-way ANOVA. To compare pSTAT levels in monocytes from normotensive and hypertensive subjects, we employed two-way ANOVA with Student Newman Keuls post hoc test. Categorical data were analysed using *χ*^2^ analysis. *P* values are reported in the figures and were considered significant when <0.05. Power analyses for the various experiments are provided in Supplementary material online, *Table S1*.

**Table 1:** Clinical characteristics of patients studied for comparison of circulating monocytes (*n*=132)

	NT	HTN well controlled	HTN poorly controlled	*P* value (ANOVA or *χ*^2^)
Age	58.1 ± 12	59.8 ± 10.9	62 ± 10	NS
Race (W/B)	20/0	52/0	60/0	–
Sex (M/F)	13 Jul	24/28	27/33	NS
BMI	26.1 ± 2.7	28.8 ± 5.7	28.0 ± 4.7	NS
SBP	120 ± 10	127 ± 8	147 ± 13	<0.001
DBP	77 ± 10	81 ± 7	90 ± 8	<0.001
Cholesterol	5.6 ± 1.4	5.2 ± 1.5	5.5 ± 1.3	NS
Smoking	20 May	16/52	16/60	NS
Hypercholesterolemia	20 Nov	35/52	48/60	NS
Medications (*n*)				Pearson *χ*^2^
ACEi	3	41	55	<0.002
CCB	1	14	36	<0.01
BB	5	33	39	0.4
Diuretic	1	25	50	<0.001
ARB	0	5	5	0.7
α1B	0	2	12	<0.02
Statin	8	26	31	0.8

ACEi, angiotensin converting enzyme inhibitor; ARB, angiotensin AT1-receptor blocker; BB, beta-blocker; α1B, alpha-1 adrenergic receptor antagonist; CCB + BB, nifedipine and metoprolol; CCB + α1B, amlodipine and doxazosin.

## 3. Results

### 3.1 Hypertensive mechanical stretch in human endothelial cells promotes monocyte activation and differentiation

It has been previously reported that endothelial cells activated by zymosan, LPS or IL-1β can promote conversion of monocytes to DCs.^22^ A feature of hypertension that activates endothelial cells is increased mechanical stretch. We therefore isolated CD14^+^ monocytes from PBMCs of normotensive humans and co-cultured these with HAECs undergoing 5% or 10% mechanical stretch for 48 h as shown by the illustration in *Figure 1A*. The intermediate monocyte CD14^++^CD16^+^ population (*Figure 1B* and *C*) and the CD14^++^CD209^+^ population (*Figure 1B* and *D*) were significantly increased in the co-cultures with HAECs undergoing 10% compared with 5% stretch. The non-classical monocyte population defined as CD14^low^CD16^++^ displayed a trend to increase in response to 10% endothelial stretch (*Figure 1E*). The macrophage population defined as CD14^+^CD163^+^ (*Figure 1F*) and the classical monocyte population defined as CD14^++^CD16^−^ (*Figure 1G*) were not different between experimental groups. Another marker of monocyte activation and DC development is CD83^36^; however, only a few monocytes expressed CD83^+^ after co-culture (*Figure 1H*) and these were not changed by endothelial stretch. Intermediate levels of endothelial stretch ranging from 6% to 8% failed to alter the phenotype of human monocytes (see Supplementary material online, *Figure S1A*). There was also no difference in the percent of live cells between the 5% and 10% stretch (see Supplementary material online, *Figure S1B*). In keeping with the state of monocyte activation, 10% endothelial stretch promoted a striking upregulation of monocyte mRNA for the cytokines IL-6, IL-1β, IL-23, TNFα, and CCL4 compared with 5% stretch (*Figure 1I*). In contrast, stretch did not affect monocyte expression of TGFβ-1, MMP8, CCL2, IL-18, or CD168 (see Supplementary material online, *Figure S2*).

It has been reported that monocytes can enter tissues and re-emerge into the circulation without differentiation into macrophages or DCs.^34^ These cells can transport antigen to lymph nodes and have enhanced ability to drive T-cell proliferation. To determine if endothelial stretch conveys this property to monocytes, we obtained T cells from the same monocyte donors after their monocytes had been exposed to endothelial cell stretch for 48 h. These autologous T cells were labelled with CFSE and co-cultured with monocytes for 7 days and their proliferation was examined by CFSE dilution. As shown in *Figure 1J*, monocytes previously exposed to 10% stretched endothelial cells for 48 h exhibited an enhanced ability to drive CD4^+^ and CD8^+^ T-cell proliferation when compared with the cells from the 5% stretched endothelial cell cultures.

### 3.2 Hypertensive mechanical stretch on endothelial cells promotes STAT3 activation in co-cultured monocytes

**Figure 2:**
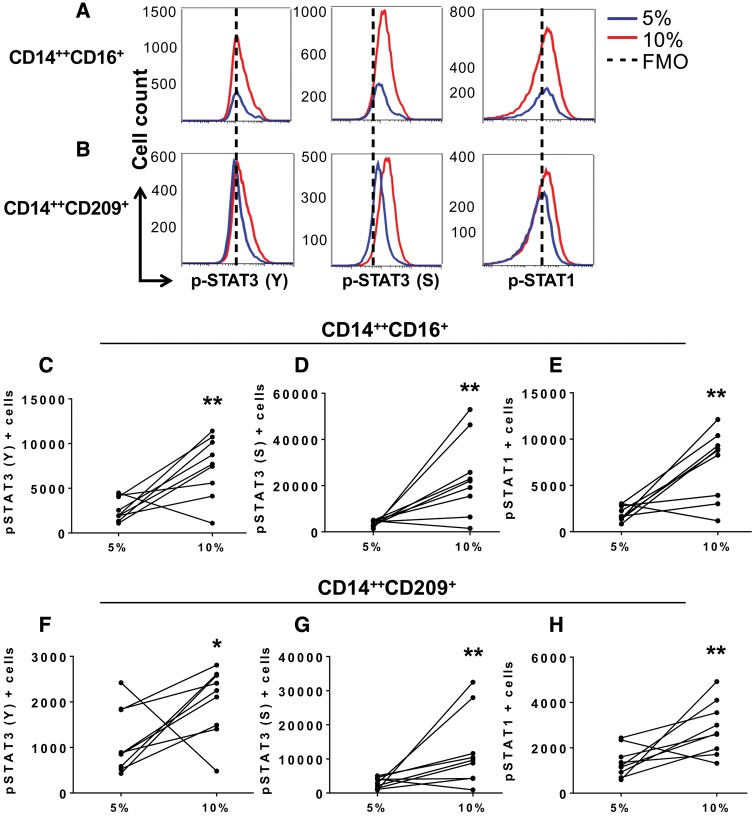
Effect of endothelial stretch on STAT3 activation in co-cultured monocytes. Human CD14^+^ monocytes were isolated from buffy coats of normal human volunteers and cultured with HAECs exposed to 5% or 10% cyclical stretch. (*A*) Representative flow cytometry plots are shown for intracellular staining of STAT3 phosphorylation in the tyrosine (Y) 705 and the serine (S) 727 and STAT1 phosphorylation in the Y701 in the CD14^++^CD16^+^ intermediate monocytes and (*B*) the CD14^++^CD209^+^ cells in the 5% stretch (blue), 10% stretch (red), and the dashed line represents FMO control. (*C*–*E*) Changes in numbers of intermediate monocytes between 5% and 10% endothelial cell stretch expressing pSTAT3 (Y), pSTAT3 (S), and pSTAT1 are depicted by connected lines. (*F*–*H*) Changes in numbers of CD14^++^CD209^+^ cells expressing pSTAT3 (Y), pSTAT3 (S), and pSTAT1 between 5% and 10% endothelial cell stretch. Comparisons were made using one-tail paired *t*-tests (*n* = 9, *P <* *0.05, **P <* *0.01).

Increased expression of IL-6, IL-1β, and IL-23 resemble a cytokine response typical of STAT3 signalling.^37^ In addition, STAT3 activation has been identified as a checkpoint for FLT-3-regulated DC development.^38^ We therefore sought to determine whether STAT3 plays a role in monocyte activation and differentiation when exposed to HAECs undergoing stretch. STAT3 activation occurs upon phosphorylation of tyrosine (Y) 705 and/or serine (S) 727. When activated, STAT3 can also form a heterodimer with STAT1. Using intracellular staining we found that the CD14^++^CD16^+^ intermediate (*Figure 2A*, *C*–*E*) and the CD14^++^CD209^+^ populations (*Figure 2B*, *F*–*H*) had a significant increase in pSTAT3 (Y705), pSTAT3 (S272), and pSTAT1 when cultured with endothelial cells undergoing 10% stretch. We also observed an increase in STAT3 and STAT1 phosphorylation in cells that remained CD14^++^CD16^−^, but not in the non-classical monocytes (see Supplementary material online, *Figure S3A* and *B*). Given that both STAT3 and STAT1 are activated in monocytes that underwent transformation, we considered the possibility that this involved heterodimerization of the two STAT isoforms, however, we were unable to detect association of the two using co-immunoprecipitation (see Supplementary material online, *Figure S3C*). Moreover, we were unable to detect fluorescence resonance energy transfer between STAT1 and STAT3 using flow cytometry-based method (data not shown).

### 3.3 STAT3 plays a role in monocyte differentiation and activation during hypertensive mechanical stretch of endothelial cells

**Figure 3:**
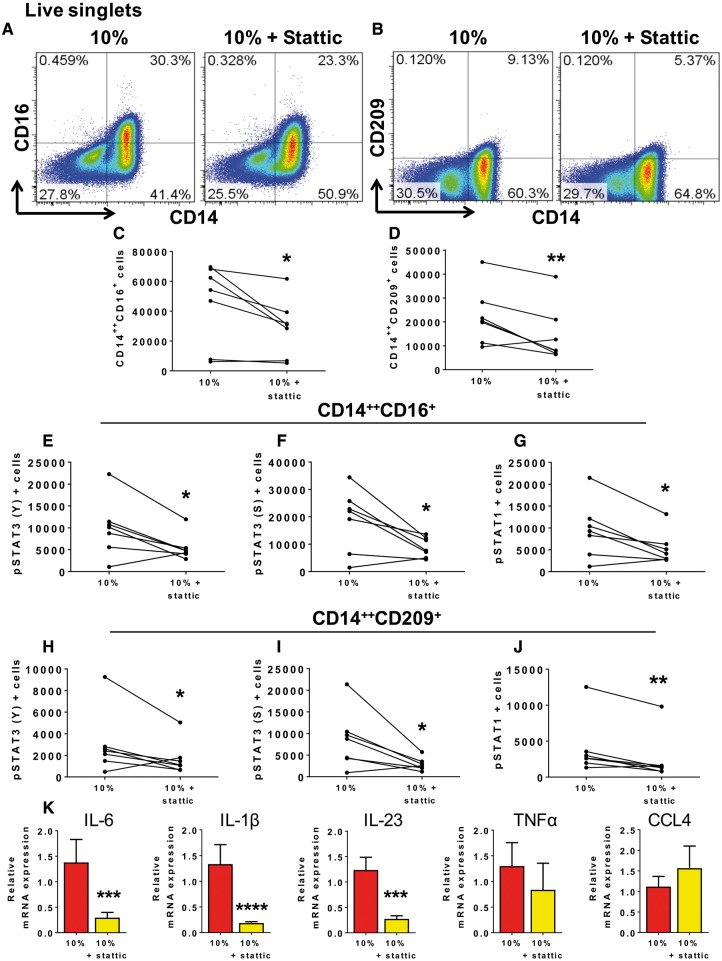
STAT3 contributes to monocyte differentiation and activation during hypertensive mechanical stretch of endothelial cells. Human CD14^+^ monocytes were isolated from PBMCs of normal human volunteers and cultured with HAECs exposed to 10% or 10% stretch plus STAT3 inhibitor (5 µM), Stattic, for 48 h. (*A*) Flow cytometry gating examples are shown for the CD14^++^CD16^+^ intermediate monocyte population and (*B*) the CD14^++^CD209^+^ cells. (*C*) Individual data point for the effect of Stattic on the number intermediate monocytes and (*D*) CD14^++^CD209^+^ cells for each subject. (*E*–*G*) Effect of Stattic on total number of cells expressing pSTAT3 (Y), pSTAT3 (S), and pSTAT1 within the intermediate monocyte population and (*H*–*J*) the CD14^++^CD209^+^ population. A total of *n* = 7 participants per group were used. (*K*) Relative monocyte mRNA expression of IL-6, IL-1β, IL-23 (10%, *n*= 11; 10% + Stattic, *n* = 9), TNFα (*n* = 7), and CCL4 (*n* =4) in monocytes. Comparisons were made using one-tail paired *t*-tests (*P <* *0.05, **P <* *0.01, ***P <* *0.001, ****P <* *0.0001).

To determine a specific role of STAT3 in differentiation of monocytes during stretch, we employed Stattic, a non-peptidic small molecule that selectively inhibits the function of the STAT3 SH2 domain.^39^ Addition of Stattic to the cell culture reduced formation of the CD14^++^CD16^+^ intermediate monocyte population (*Figure 3A* and *C*) and CD14^++^CD209^+^ DC population in response to 10% stretch (*Figure 3B* and *D*). Likewise, Stattic reduced pSTAT3 (Y), pSTAT3 (S), and pSTAT1 in the CD14^++^CD16^+^ intermediate (*Figure 3E*–*G*) and the CD14^++^CD209^+^ monocyte populations (*Figure 3H*–*J*). Furthermore, we found that addition of Stattic to monocyte-HAEC cultures undergoing 10% stretch reduced upregulation of mRNA for the cytokines IL-6, IL-1β, and IL-23 (*Figure 3K*).

**Figure 4:**
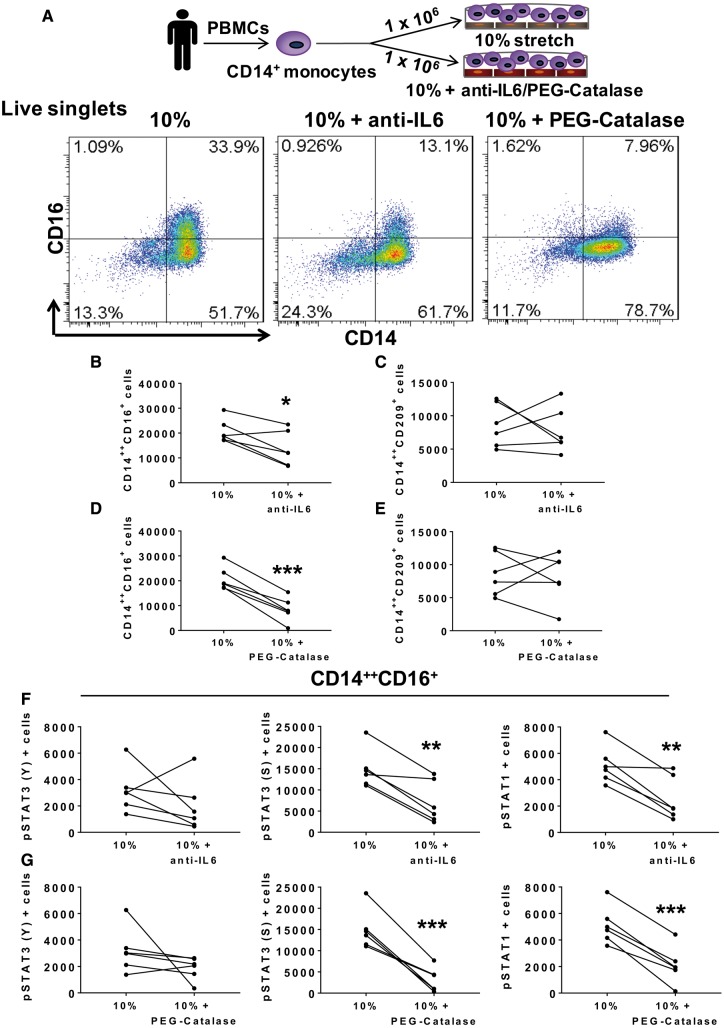
IL-6 and hydrogen peroxide play a role in monocyte transformation and activation. Human CD14^+^ monocytes were isolated from PBMCs of normal human volunteers and cultured with HAECs exposed to 10% stretch, 10% plus anti-IL-6 neutralization antibody (10 µg/mL) or 10% plus PEG-Catalase (500 U/mL) for 48 h. (*A*) Schematic of methods and flow cytometry gating representatives are shown for the CD14^++^CD16^+^ intermediate monocyte population exposed to 10%, 10% + anti-IL-6, and 10% + PEG-Catalase. (*B*) Effect of anti-IL-6 on the total number of intermediate monocytes and (*C*) the CD14^++^CD209^+^ cells for each subject. (*D*) Effect of PEG-Catalase on total number of intermediate monocytes and (*E*) the CD14^++^CD209^+^ cells for each participant. (*F*) Effects of anti-IL-6 and (*G*) PEG-Catalase on number of cells expressing pSTAT3 (Y), pSTAT3 (S), and pSTAT1 within the intermediate monocyte population for each subject. Data with and without these interventions for each subject are shown by the connected lines. A total of *n* = 6 participants per group and per experimental treatment were used. Comparisons were made using one-tail paired *t*-tests (*P <* *0.05, **P <* *0.01, ***P <* *0.001).

Next, we sought to determine mechanisms by which endothelial cells undergoing stretch could activate monocytes and promote STAT3 phosphorylation in adjacent monocytes. Others have reported that stretch stimulates expression of IL-6 by endothelial cells, and we confirmed a two-fold increase in IL-6 protein production by HAECs undergoing cyclical stretch (see Supplementary material online, *Figure S4*). IL-6 has been shown to both stimulate STAT3 activation and to be produced in response to STAT3 in a feed-forward fashion.^40^ Addition of an IL-6 neutralizing antibody to the endothelial/monocyte co-cultures markedly reduced formation of intermediate monocytes (*Figure 4A* and *B*), while having no effect on the CD14^++^CD209^+^ population (*Figure 4C*).

STAT3 can also be activated by reactive oxygen species (ROS), including hydrogen peroxide.^37^ ROS, in turn can stimulate IL-6 production by the endothelium.^41^ Because increased endothelial stretch can stimulate ROS formation, we performed additional experiments using Tempol, a superoxide dismutase mimetic, or polyethylene glycol (PEG)-Catalase, to scavenge hydrogen peroxide. While Tempol had no effect (data not shown), we found that PEG-Catalase markedly reduced formation of intermediate monocytes in response to 10% endothelial stretch (*Figure 4D*). Like anti-IL-6, PEG-Catalase did not inhibit formation of the CD209 population (*Figure 4E*). In keeping with these results with anti-IL-6 and PEG-Catalase, we found that these interventions also inhibited pSTAT3 (S727) and pSTAT1 (Y701) and exhibited a trend to inhibit pSTAT3 (Y705) (*Figure 4F*–*G*) within the intermediate monocytes.

**Figure 5:**
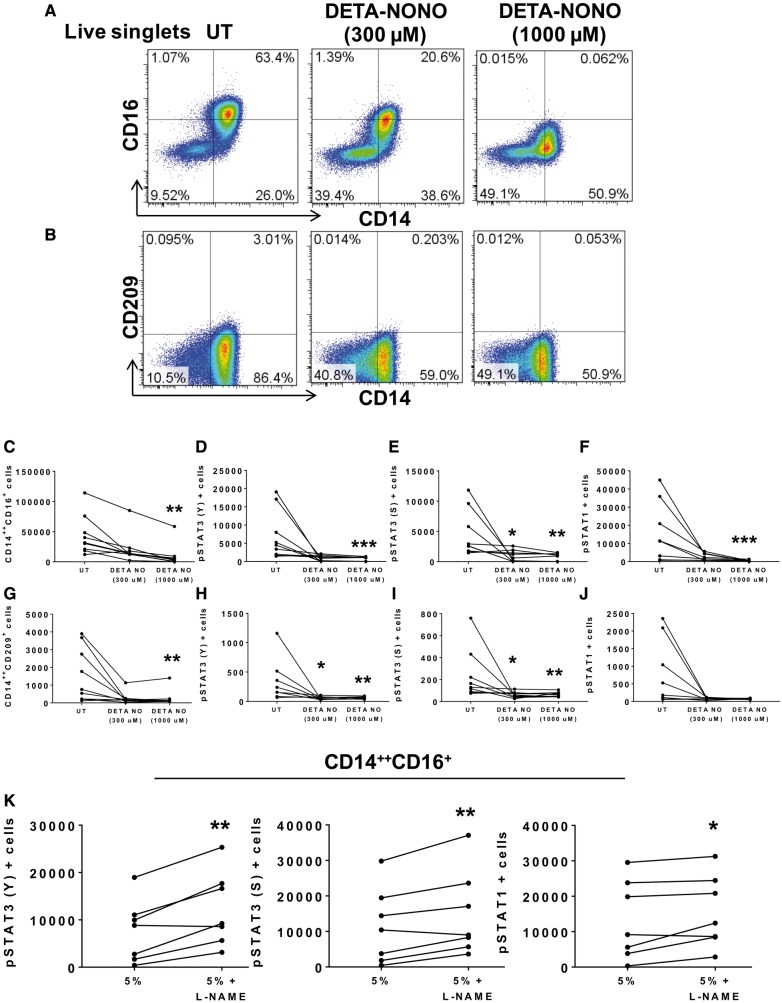
Exposure of monocytes to NO donor inhibits human monocyte conversion and activation to its derived populations. Human CD14^+^ monocytes were isolated from buffy coats of normal human volunteers and cultured alone in untreated (UT) conditions, DETA-NONOate (DETA-NONO), an NO donor, at 300 µM or DETA-NONO at 1000 µM concentrations in static conditions for 48 h. (*A*) Flow cytometry representatives are shown for the CD14^++^CD16^+^ intermediate monocyte and (*B*) the CD14^++^CD209^+^ population. (*C*) Values for each subject without and with DETA-NONO are shown for the total number of cells from the CD14^++^CD16^+^ intermediate monocytes and for the total number of cells expressing (*D*) pSTAT3 (Y), (*E*) pSTAT3 (S), and (*F*) pSTAT1 within this population. (*G*) Effect of DETA-NONO on the number of CD14^++^CD209^+^ cells and the expression of (*H*) pSTAT3 (Y), (*I*) pSTAT3 (S), and (*J*) pSTAT1 for each subject. A total of *n*= 9 participants per group were used for these experiments. (*K*) Human CD14^+^ monocytes were cultured with HAECs exposed to 5% stretch or 5% plus NO synthase inhibitor, L-NAME (1000 µM) for 48 h. The number of CD14^++^CD16^+^ intermediate monocyte population expressing pSTAT3 (Y), pSTAT3 (S), and pSTAT1 for each subject are connected by lines. A total of *n* = 7 participants per group were used. For (*C*–*J*) the nonparametric Friedman’s test followed by Dunn’s multiple comparison tests was employed. For (*K*) a one-tailed paired *t*-tests was used (*P <* *0.05, **P <* *0.01, ***P <* *0.001).

Increased endothelial stretch has also been shown to uncouple the endothelial NO synthase and to reduce stimulatory phosphorylation of endothelial NO synthase in endothelial cells.^31^^,^^42^ Likewise NO has been shown to suppress IL-6-induced STAT3 activation in ovarian cancer cells.^43^ We therefore hypothesized that a loss of bioavailable NO might promote STAT3 activity. In keeping with this hypothesis, we found the NO donor DETA NONOate (DETA NONO) dramatically reduced formation of intermediate monocytes (*Figure 5A* and *C*) and the activation of STAT3 (Y), STAT3 (S), and STAT1 when added to monocytes cultures in the absence of endothelial cells (*Figure 5D*–*F*). Furthermore, addition of this NO donor also reduced formation of CD14^++^CD209^+^ cells (*Figure 5B* and *G*) and activation of STAT3 (Y05), STAT3 (S727), and STAT1 (*Figure 5H*–*J*). We further found that addition of the NO synthase inhibitor L-NAME to monocytes undergoing 5% stretch significantly increased pSTAT3 (Y705), pSTAT3 (S272), and pSTAT1 levels in intermediate monocytes (*Figure 5K*).

We considered the possibility that LECT2, a ligand for CD209, might be released by endothelial cells, however we were unable to detect LECT2 released from HAECs undergoing either 5% or 10% stretch by ELISA (data not shown). We were also unable to detect release of HMBG-1 chromatin binding protein, which has been shown to activate STAT3,^44^ from stretched endothelial cells (data not shown).

We also examined the hypothesis that monocytes might adhere to the endothelium and themselves undergo cyclical stretch. In keeping with this, we found that 10% stretch increased ICAM-1 expression on endothelial cells and increased adhesion of monocytes to the HAECs compared to the 5% stretch controls (see Supplementary material online, *Figure S5A*). We also employed confocal microscopy with Z stacking to interrogate the endothelial layer and the subendothelial collagen to visualize CD31^+^, CD14^+^, and CD83^+^ cells. Using this approach, we observed CD14^+^ monocytes on the surface of endothelial cells in the co-culture (see Supplementary material online, *Figure S5B*). Moreover, CD83^+^ cells were observed on the surface of the CD31^+^ endothelial cells exposed to 10% stretch, while none were detected in cultures exposed to 5% stretch. We observed no monocyte or CD83 expressing cells in the collagen/gelatin sub-endothelial space in either the 5% or 10% stretch experiments.

To determine if stretch could directly activate monocytes in the absence of endothelial cells, we cultured monocytes in arginylglycylaspartic acid (RGD)-covered Pronectin^®^ or Collagen I coated stretch plates without the presence of HAECs, and exposed these to either 5% or 10% levels of stretch. By visualizing cells at 24 h, we found that both Pronectin^®^ and Collagen I coated stretch plates promoted monocyte adhesion (see Supplementary material online, *Figure S5C*). We examined monocyte phenotypes following 48 h of stretch on these plates, and found that neither 5% or 10% stretch in the absence of endothelial cells supported monocyte transformation (see Supplementary material online, *Figure S5D*).

### 3.4 Hypertension affects the distribution of circulating mononuclear cells in humans

**Figure 6:**
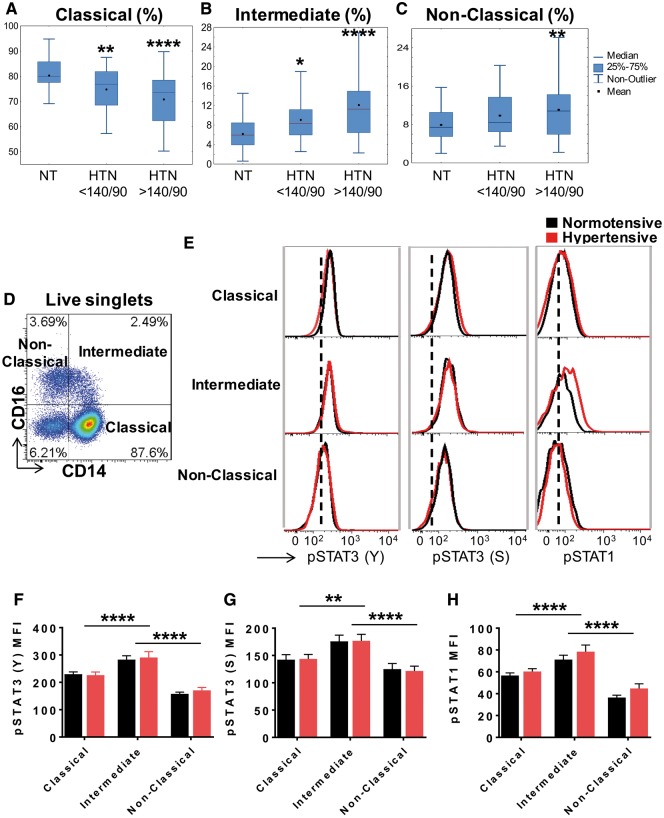
Hypertension affects the distribution of circulating monocytes in humans. (*A*) A cohort of normotensive (*n* = 20), mildly hypertensive (systolic BP ∼130–140 mmHg, *n* = 52), and severely hypertensive (systolic BP >140 mmHg, *n* = 60) subjects were recruited and flow cytometry was used to analyse various monocyte populations. (*A*) Mean values are shown for the percent of positive CD14^++^CD16^−^ classical, (*B*) CD14^++^CD16^+^ intermediate and (*C*) CD14^low^CD16^++^ non-classical monocytes comparing normotensive, mild, and severely hypertensive subjects. Data were analysed using one-way ANOVA. (*D*) Representative flow cytometry dot plots showing classical, intermediate, and non-classical subset distribution are shown. (*E*) Histograms showing the CD14^++^CD16^−^ classical, CD14^++^CD16^+^ intermediate, non-classical monocyte population comparing normotensive (black) and hypertensive (red) subjects. The dashed line represents the Mean fluorescent intensity (MFI) for the FMO control. (*E*) MFI for pSTAT3 (Y), pSTAT3 (S) (*F*), and pSTAT1 (*G*) in various monocyte subgroups in both normotensive (*n* = 15) and hypertensive (*n* = 12) subjects. Two-way ANOVA with Student Newman Keuls post hoc test was used (*P <* *0.05, **P <* *0.01, ***P <* *0.001, ****P <* *0.0001).

In additional experiments, we sought to determine if hypertension is associated with an alteration of the phenotype of circulating monocytes. The demographics of the 132 subjects included for this analysis are shown in *Table 1*. Using a gating strategy published previously,^35^ we found that there is a progressive decline in the classical monocytes and a concomitant increase in the percent of intermediate and non-classical monocytes with increasing levels of hypertension (*Figure 6A*–*C*). In additional studies we sought to determine if intermediate monocytes or non-classical monocytes exhibit evidence of STAT activation. We recruited an additional 15 normotensive and 12 hypertensive subjects for this analysis. The demographics of these subjects are shown in *Table 2* and a representative dot plot for the different monocyte populations is shown in *Figure 6D*. Example histograms for STAT phosphorylation in normotensive and hypertensive individuals are shown in *Figure 6E*. Intermediate monocytes exhibited increased phosphorylation of STAT3 Y705, STAT3 S727, and STAT1 Y701 compared with other monocyte populations (*Figure 6F*–*H*). No differences between normotensive and hypertensive groups were observed.

**Table 2:** Demographics of patients studied for analysis of phospho-STAT in circulating monocytes

	Normotensive	*P*	Hypertensive
*n*	15		12
Age (years)	44.8±3.9	NS	51.3±2.6
Race (W/B)	15/0	NS	3 Sep
Gender (F/M)	4-Nov	NS	3 Sep
BMI (Kg/m^2^)	26.6±1.3	<0.03	34.7±2.9
SBP (mmHg)	110.8±3.7	<0.00003	140.5±4.1
DBP (mmHg)	64.4±1.6	<0.003	78.7±3.6
Drug Rx			
None	15		2
ACEi	0		4
ARB	0		1
HCTZ+ACEi	0		2
HCTZ+ARB	0		1
CCB+BB	0		1
CCB+α1B	0		1

ACEi, angiotensin converting enzyme inhibitor; ARB, angiotensin AT1-receptor blocker; HCTZ, hydrochlorothiazide; BB, beta-blocker; α1B, peripheral alpha-1 adrenergic receptor antagonist; CCB + BB, nifedipine and metoprolol; CCB + α1B, amlodipine and doxazosin.

### 3.5 Angiotensin II-induced hypertension in WT C57Bl/6 mice promotes accumulation of myeloid cells containing activated STAT3 in the kidney and aorta

**Figure 7:**
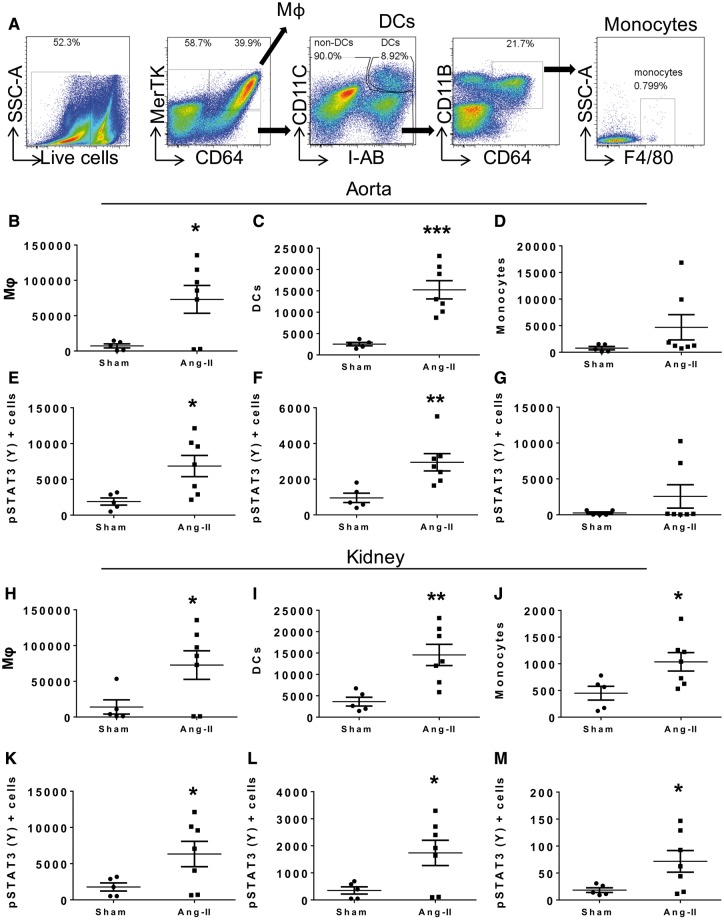
Angiotensin II-induced hypertension in wildtype C57Bl/6 mice promotes an increase in STAT3 phosphorylation in the immune cells from kidney and aorta. (*A*) Representative flow cytometry dot plots showing the gating strategy to identify macrophages, DCs and monocytes from C57Bl/6 wildtype mice infused with Ang II (490 ng/kg/min) or sham for 6 days. (*B*–*D*) Mean values of absolute numbers of indicated cell types per thoracic aorta. (*E*) Mean values of pSTAT3 (Y) expression within the macrophage (Mφ), (*F*) DC and (*G*) monocyte populations per thoracic aorta. (*H*–*J*) Mean values of absolute numbers of indicated cell types per kidney. (*K*) Mean values of pSTAT3 (Y) expression within the macrophage, (*L*) DC and (*G*) monocyte populations per kidney. A total of *n* = 5, sham, and *n* =7, Ang II, treated mice per group were used. One-tail unpaired *t*-test was employed (*P <* *0.05, **P <* *0.01, ***P <* *0.001).

**Figure 8:**
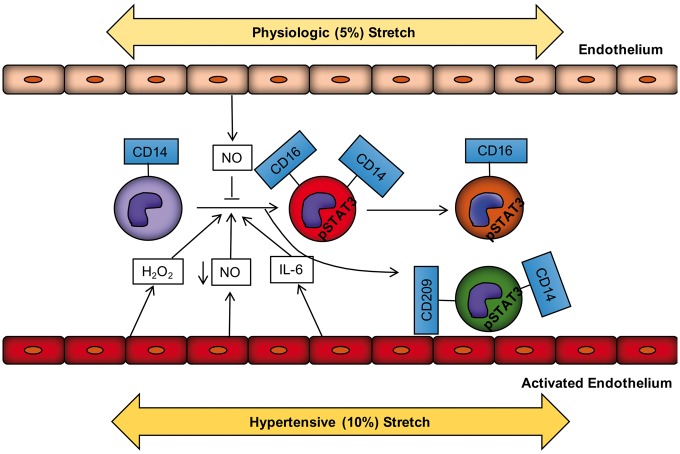
Increased endothelial stretch promotes monocyte transformation and activation. Under physiological stretch, the endothelium releases nitric oxide that prevents monocyte activation and transformation. In hypertension, increased vascular stretch promotes endothelial activation. The activated endothelium in turn releases ROS including hydrogen peroxide, cytokines including IL-6 and exhibits reduced NO bioavailability. These events initiate STAT3 activation in the monocytes and promote transformation of the classical monocyte into an intermediate and subsequently a non-classical monocyte as well as a cells bearing the CD209 DC marker.

In subsequent experiments, we sought to determine the role of hypertension on monocyte transformation *in vivo*. We induced hypertension in C57Bl/6 mice by infusion of Ang II (490 ng/kg/min) for 6 days and analysed single cell suspensions of the kidney, aorta, spleen, and periaortic lymph nodes for the presence of monocytes, macrophages (MΦ) and DCs using a gating strategy that effectively allows discrimination of these cells (*Figure 7A*). We found a significant increase in the total number of macrophages (*Figure 7B*) and DCs (*Figure 7C*) and a trend of an increase in the monocytes (*Figure 7D*) within the aorta of Ang II treated mice. This increase in cell number was accompanied by an increase in STAT3 (Y705) activation (*Figure 7E* and *F*) and a trend of an increase in the monocyte population (*Figure 7G*). We also found an increase in macrophages, DCs and monocytes in the Ang II treated mice within the kidneys (*Figure 7H*–*J*) and an increase in STAT3 activation in each of these populations (*Figure 7K*–*M*). In the lymph nodes, macrophages were increased in response to Ang II infusion, while there was no change in monocytes or DCs (see Supplementary material online, *Figure S6A*). We also observed an increase in STAT3 activation of monocytes in lymph nodes. There were no major changes in these populations and in STAT3 activation in the spleen of Ang II-treated mice (see Supplementary material online, *Figure S6B*). To determine the location of monocytes infiltrating the aorta after 2 weeks of Ang II infusion in C57Bl/6 mice, we performed immunofluorescence of aortic sections for F4/80 (red), pSTAT3 (Y705, green), and DAPI. We found that after 2 weeks of Ang II infusion, monocytes and macrophages localize in the perivascular fat and adventitia and these co-localize with pSTAT3 (Y705) (see Supplementary material online, *Figure S7*) (*Figure 8*).

## 4. Discussion

In this study, we demonstrate that exposure of human monocytes to endothelial cells undergoing 10% mechanical stretch increases differentiation into CD14^++^CD16^+^ intermediate monocytes and CD14^++^CD209^+^ cells. We further show that monocytes cultured with endothelial cells exposed to hypertensive mechanical stretch markedly increase expression of IL-6, IL-1β, IL-23, CCL4, and TNFα and have enhanced ability to drive proliferation of T cells from the same human donor. In addition, we found that endothelial cells undergoing hypertensive mechanical stretch stimulate an increase in pSTAT3 (Y), pSTAT3 (S), and pSTAT1 within these monocyte populations. Inhibition of STAT3 by Stattic prevented conversion of monocytes into the intermediate and the DC phenotype and normalized the cytokine production of these monocytes cultured with endothelial cells undergoing 10% stretch. Our data implicate a role of hydrogen peroxide and IL-6 as mediators of monocyte differentiation. We also show that hypertension is associated with an increase in the percentage of circulating intermediate and non-classical monocytes and that circulating intermediate monocytes exhibit higher levels of pSTAT3 (Y), pSTAT3 (S), and pSTAT1. Finally, the tissue infiltrating monocytes, macrophages, and DCs exhibited an increase in phosphorylated STAT3 in hypertensive mice. Thus, altered mechanical forces affecting the endothelium can modify monocyte differentiation and activation and likely contribute to immune activation in hypertension.

Intermediate monocytes are the least characterized of the monocyte subtypes in humans, however, these cells have been implicated in inflammatory diseases such as Kawasaki disease,^45^ rheumatoid arthritis,^15^ sepsis,^46^ HIV,^47^ acute heart failure, and coronary artery disease.^48^ Using deuterium labelling in humans, Patel *et al.* recently showed a sequential transition of classical monocytes that emerge from the bone marrow to the intermediate and subsequently the non-classical phenotype.^11^ Compared with classical monocytes, intermediate monocytes exhibit enhanced phagocytosis, produce higher levels of ROS and inflammatory mediators such as TNFα and IL-1β.^12^^,^^49^^,^^50^ They also have the highest expression of the major histocompatibility complex class II antigens, including histocompatibility leukocyte antigen (HLA)-DR, -DP, and -DQ indicating that they also possess antigen presentation functions.^51^ Thus, the presence of high numbers of intermediate monocytes in humans with hypertension likely has pathophysiological implications.

Our data are compatible with findings by Randolph *et al.* showing that activated endothelial cells can modify monocyte phenotype. They demonstrated that monocytes that transmigrate the endothelium convert to macrophages that remain in the subendothelial space or DCs expressing CD83 that reverse transmigrate the endothelium.^22^ In a subsequent study, this group also showed that a subpopulation of CD16^+^ monocytes have a propensity to reverse transmigrate the endothelium and, upon doing so, increase their expression of CD86^+^ and HLA-DR. In keeping with this, the authors showed these monocytes potently induced allogenic T-cell proliferation.^23^ The investigators assumed cells had transmigrated the endothelial monolayer if they were not removed by washing the monolayer 2 h after addition of the cells. In our study, we found that 10% stretch markedly enhanced monocyte binding to the endothelial surface. Our analysis, however, did include cells potentially within the subendothelial space and it also demonstrated the presence of macrophage-like cells expressing the CD163 marker; however, these were not altered by the degree of endothelial stretch.

In these experiments, we not only examined intermediate and non-classical monocytes, but also CD14^+^ cells bearing the markers CD209 and CD83. These have been employed as DC markers, but can be expressed on a variety of activated myeloid cells. CD209 is a C-type Lectin receptor that promotes the production of IL-1β and IL-23 that can ultimately skew T cells to a T_H_17 phenotype. Levels of CD209 on whole leukocytes correlate with disease severity in Behçet’s Disease.^52^ LECT2 acts on CD209 to induce c-Jun N-terminal kinase (JNK) signalling in monocytes and endothelial cells.^20^ CD209 forms a complex with TLR4 and promotes NFκB activation in response to oxidized low-density lipoprotein (LDL) confirming the role of CD209 in innate immunity and antigen presentation.^53^ While we were not able to detect LECT2 levels in our co-culture system, the increase in CD209 on monocytes could arm these cells to respond to signals such as LECT2 or oxidized LDL *in vivo*. CD83 is a type-I lectin transmembrane protein expressed on monocyte-derived DCs and small subsets of other immune cells.^54^ The presence of CD83 on DCs enhances their ability to evoke T-cell calcium transients and proliferation.^36^ Thus, surface expression of CD209 and CD83 may have functional consequences in enhancing immunogenicity of monocyte-derived cells in hypertension.

There are a number of factors in the hypertensive milieu that could activate both endothelial cells and monocytes, including cytokines and oxidative stress.^55^^,^^56^ In this investigation, we focused on the role of increased endothelial stretch, which is known to stimulate endothelial cell cytokine production, ROS formation and expression of adhesion molecules.^31^ The vessels affected by stretch in hypertension likely vary depending on the duration of the disease. Early in hypertension, there is increased stretch of large arteries; however, as hypertension is sustained, these proximal vessels become stiff, leading to propagation of the forward pulse wave velocity into smaller resistance arteries.^57^ Our present data therefore might explain how increased cyclic stretch in small vessels could promote immune activation. Consistent with this, we have previously shown that aortic stiffening precedes renal accumulation of T cells, monocyte and macrophages ultimately leading to renal dysfunction.^58^

In these experiments, we found evidence for STAT activation in intermediate monocytes of humans and in monocytes exposed to endothelial cells undergoing 10% cyclical stretch as evidenced by phosphorylation of STAT1 at Y701 and STAT3 at S727 and Y705. Of interest, STAT3 is required for Flt3-dependent formation of DCs from bone marrow derived cells,^38^ and plays an important role in production of IL-1β, IL-6, and TNFα in macrophages of humans with coronary artery disease.^37^ We also found that exposure of monocytes to endothelial cells undergoing 10% stretch markedly enhanced mRNA expression of these cytokines and that STAT3 is activated in these cells. Inhibition of STAT3 with Stattic not only inhibited cytokine production by human monocytes, but also reduced their conversion to intermediate monocytes and CD14^++^CD209^+^ cells. Thus, STAT-signalling seems to play an important role in monocyte differentiation and activation in hypertension. In keeping with this, we also observed an increase in STAT3 activation the monocyte-derived cells in the aorta and kidney of mice with Ang II-induced hypertension. These results are compatible with findings of Johnson *et al.*, who showed that STAT3 inhibition with S3I-201 prevented Ang II-induced hypertension and vascular dysfunction *in vivo*.^59^

We made substantial efforts to identify factors released by the endothelium that would mediate STAT3 activation. STAT3 can be activated by myriad factors, including growth factors, numerous cytokines, Janus tyrosine kinase (JAK), ROS, heat shock proteins and xenobiotics.^60^ IL-6 is both upstream and downstream of STAT3 activation, and we found that endothelial cells undergoing 10% stretch produced increased amounts of this cytokine. This is compatible with prior gene profiling studies showing that cyclical stretch increases IL-6 expression in endothelial cells.^28^ We found that immune-clearing of IL-6 inhibited STAT3 activation and monocyte transformation to the intermediate phenotype suggesting a scenario in which IL-6 released by the endothelium stimulates STAT3 activation in adjacent monocytes, and ultimately greater amounts of IL-6 production by these latter cells in a feed-forward fashion. Of note, IL-6 quartiles were found to be strongly associated with the risk of developing hypertension in the Nurses Health Study,^61^ and mice lacking this cytokine are protected against Ang II-induced hypertension.^62^

We also found that PEG-Catalase, which scavenges hydrogen peroxide, prevents monocyte transformation and STAT3 activation. This finding is compatible with prior studies showing that cyclical stretch activates production of ROS by endothelial cells, initially via activation of the NADPH oxidase and subsequently from uncoupled NO synthase.^31^ In preliminary studies, we confirmed that superoxide production, as measured by detection of 2-hydroxyethidium formation from dihydroethidium, was increased in both monocytes and endothelial cells when the latter cells were exposed to 10% vs. 5% stretch (data not shown). Our data suggest that superoxide is unlikely the mediator of monocyte transformation, as the superoxide dismutase mimetic Tempol failed to prevent formation of intermediate cells or STAT3 activation. It is therefore likely that hydrogen peroxide, formed by dismutation of superoxide, mediates these effects. Hydrogen peroxide is relatively stable and thus likely to serve a paracrine-signalling role in mediating cross talk between the endothelium and adjacent monocytes. Of interest, the expression of haeme oxygenase, which has anti-oxidant properties, inversely correlates with monocyte expression of CD14 in humans.^63^

In our experimental setup, we cannot exclude the possibility that IL-6 and hydrogen peroxide also have effects on the endothelium. Activation of the IL-6 receptor has been shown to stimulate the JAK-STAT pathway that can lead to further IL-6 production.^64^ Likewise, hydrogen peroxide can activate the NADPH oxidase, which could ultimately lead to additional hydrogen peroxide production in a feed forward fashion.^65^ We have also shown that ROS released from the mitochondria can stimulate the NADPH oxidase in endothelial cells.^66^ Thus, the production of IL-6 and hydrogen peroxide in a milieu of endothelial cells and monocytes could have actions on both cell types, but ultimately lead to monocyte differentiation. 

Another potential mediator of STAT activation is loss of NO signalling. NO has been shown to inhibit STAT3 activation in ovarian cancer cells and endothelial NO bioavailability is commonly lost in hypertension and related diseases.^67^^,^^68^ Monocytes in the circulation are constantly exposed to endothelial-derived NO, however, when placed in culture, spontaneously acquire CD16. We confirmed this in our studies and found that this was associated with STAT3 phosphorylation and that the NO donor DETA-NONOate markedly inhibited monocyte transformation and STAT activation. We also found that the addition of L-NAME to monocyte-HAECs cultures undergoing 5% stretch promoted STAT activation, in a fashion similar to 10% stretch. L-NAME exposure did not cause monocyte transformation during this 48-h exposure, suggesting that other signals like IL-6 and hydrogen peroxide might also be needed for this response. In keeping with our findings in the co-culture experiments, we found that hypertensive humans have an increase in circulating intermediate and non-classical monocytes, and that that this seems dependent on their severity of hypertension. It is likely that this shift in monocyte population predisposes to further inflammatory responses, including production of cytokines and T-cell activation. Of note, we also confirmed that intermediate monocytes of both hypertensive and normotensive subjects exhibit STAT1 and STAT3 activation. This is compatible with a scenario in which intermediate monocytes consistently exhibit and likely require STAT activation for their transformation from the classical precursors, but that this transformation is higher in hypertension, perhaps due to encounter with activated endothelium.

In summary, our current study provides a previously unrecognized link between mechanical forces affecting the endothelium and activation of monocytes. This provides new insight into how the immune system can be activated in hypertension and for the first time implicates intermediate monocytes as potentially important in this disease. Intermediate monocytes are not only a biomarker of inflammation in hypertension, but their acquisition of CD16 arms them to possess cytotoxic function and to produce TNFα. The production of cytokines like IL-6, IL-23, and IL-1β can also skew T cells produce IL-17A, which we have previously shown to be involved in hypertension.^8^ It is therefore likely that altered endothelial mechanical forces have important effects on immune cell function, leading to end organ dysfunction and worsening hypertension.

## Funding

This work was supported by National Heart and Blood Institute of the National Institutes of Health Grants [R01HL039006-27, R01HL125865-04, and P01HL129941], an American Heart Association Strategically Focused Research Network Grant (14SFRN20420046) and National Heart, Lung, and Blood Institute of the National Institutes of Health, Ruth L. Kirschstein National Research Service Award (NRSA) Individual Predoctoral Fellowship to Promote Diversity in Health-Related Research (F31HL132526). The work performed in Glasgow was supported by the Engineering and Physical Sciences Research Council (EPSRC) grant EP/L014165/1 and the British Heart Foundation grant RE/13/5/30177. Dr Guzik is a recipient of the Wellcome Trust Senior Biomedical Fellowship, National Science Centre of Poland (No. 2011/03/B/NZ4/02454) and BHF Centre of Research Excellence (RE/13/5/30177). 

**Conflict of interest:** none declared.

## Supplementary Material

Supplementary Fig 1

Supplementary Fig 2

Supplementary Fig 3

Supplementary Fig 4

Supplementary Fig 5

Supplementary Fig 6

Supplementary Fig 7

Supplementary Data
