# Supplementary figures and images for: Hypertension and increased endothelial mechanical stretch promote monocyte differentiation and activation: roles of STAT3, interleukin 6 and hydrogen peroxide
Source: Cardiovasc Res. 2018 May 23;114(11):1547–63. doi: 10.1093/cvr/cvy112 (PMC6106108; doi:10.1093/cvr/cvy112)

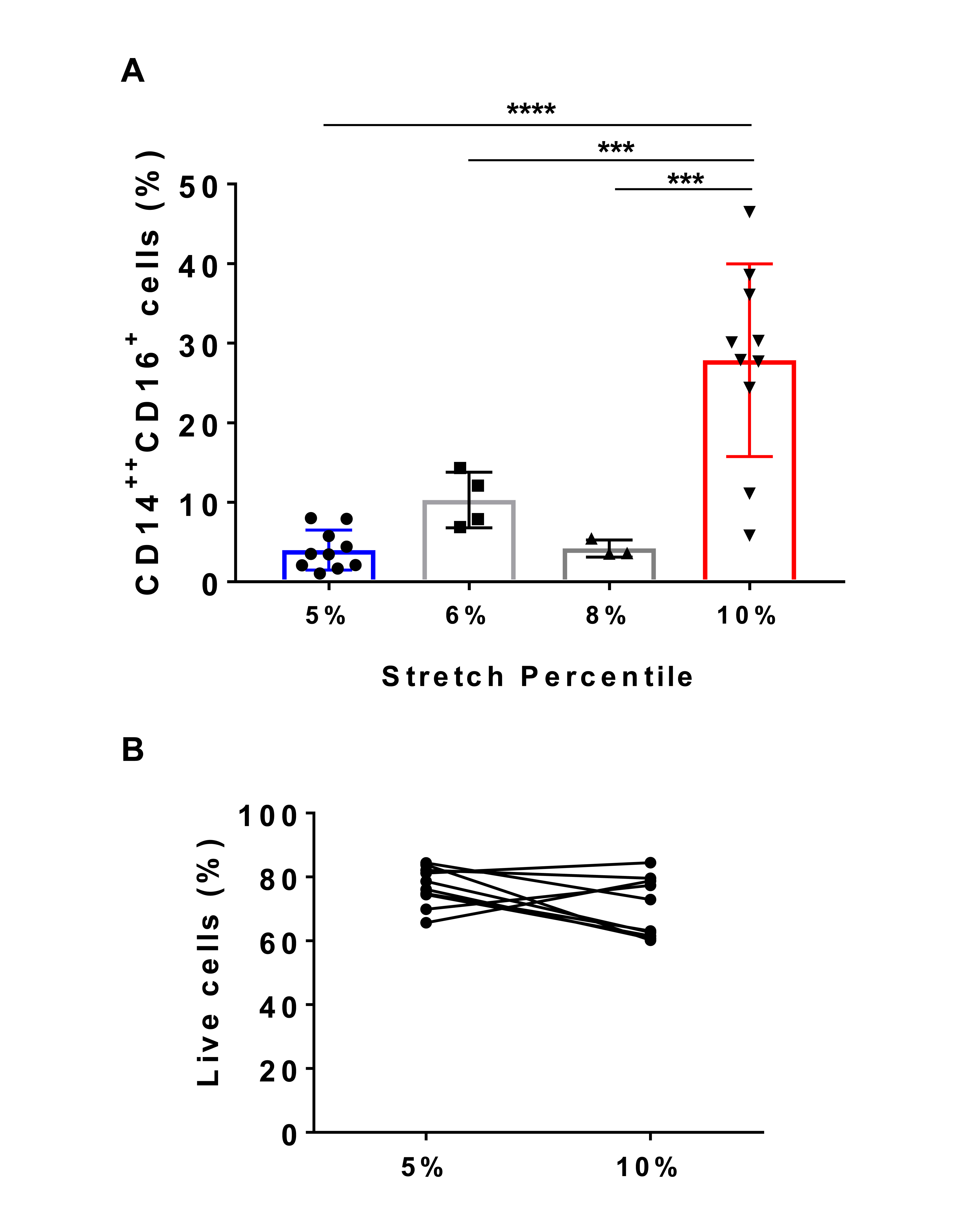

Supplement: Supplementary Fig 1 [file cvy112_supplementary_fig_1.jpeg]

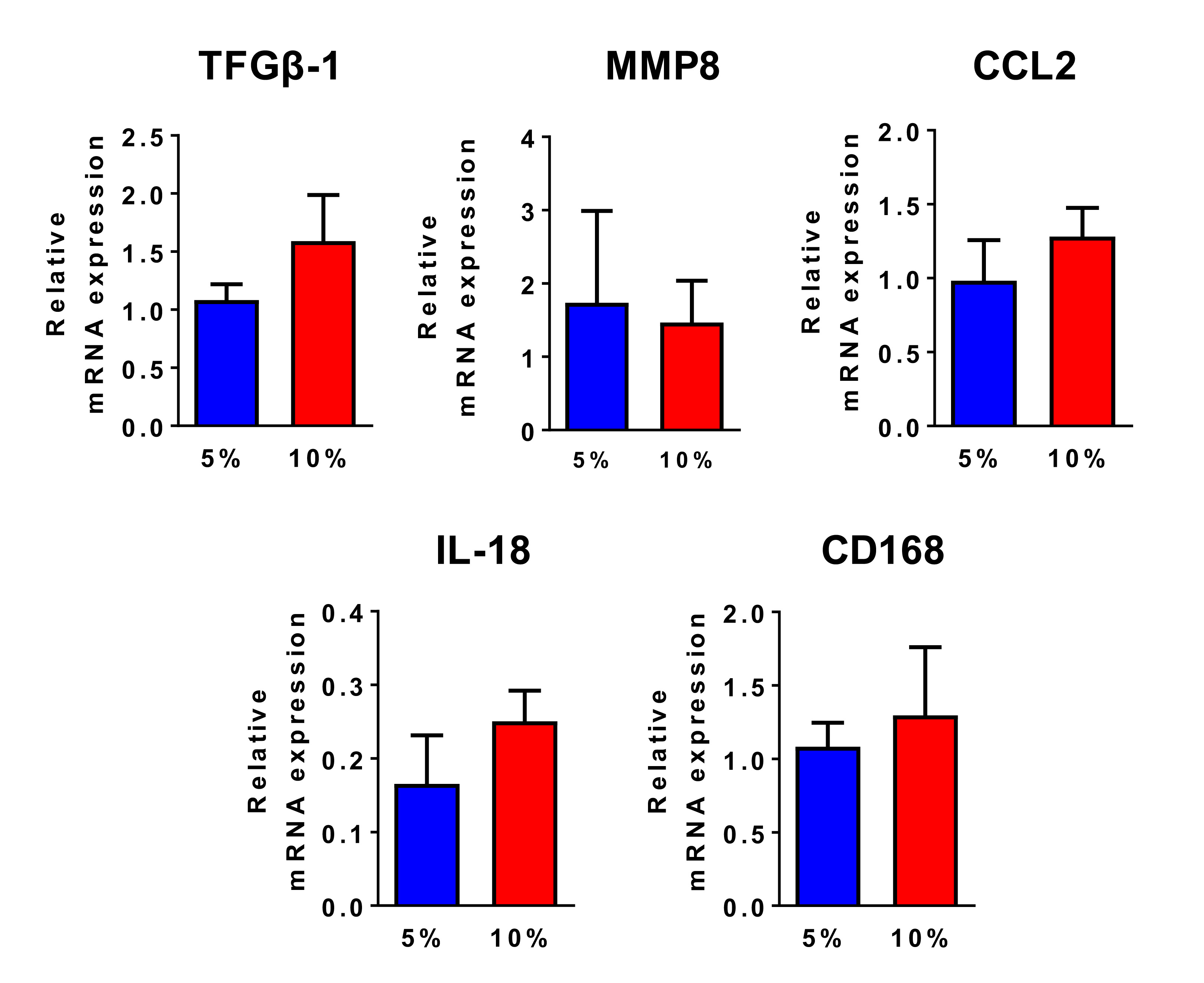

Supplement: Supplementary Fig 2 [file cvy112_supplementary_fig_2.jpeg]

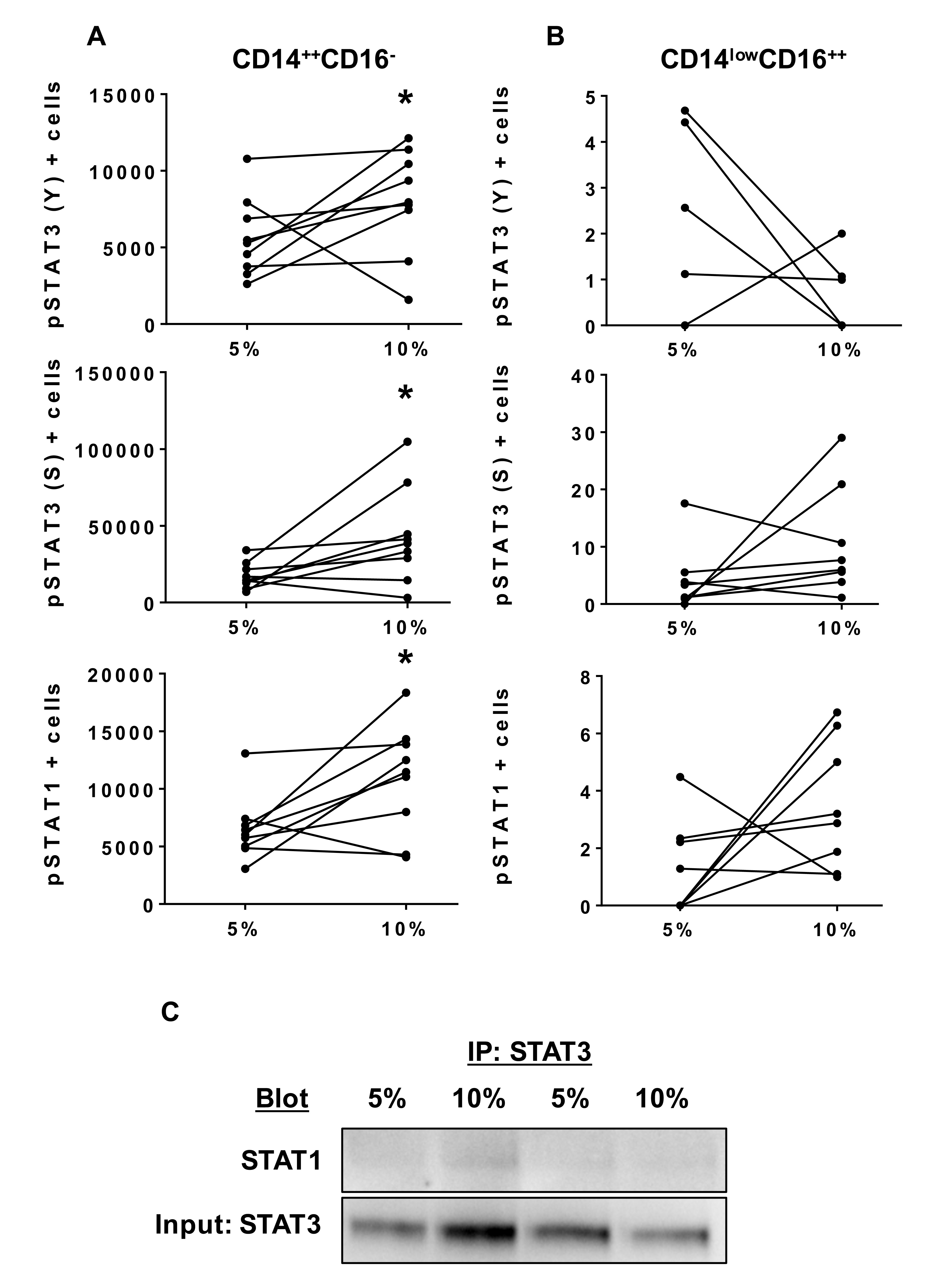

Supplement: Supplementary Fig 3 [file cvy112_supplementary_fig_3.jpeg]

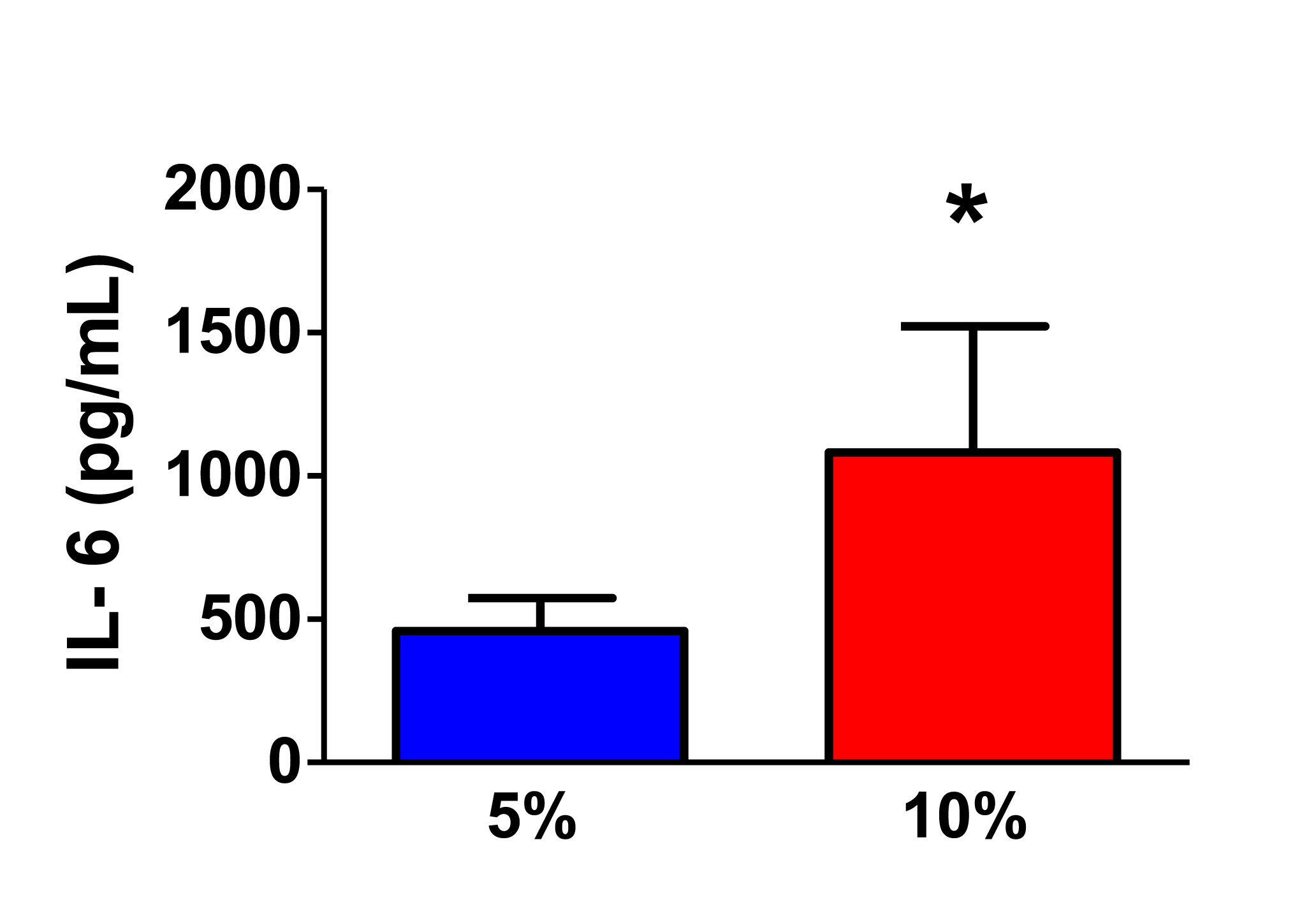

Supplement: Supplementary Fig 4 [file cvy112_supplementary_fig_4.jpeg]

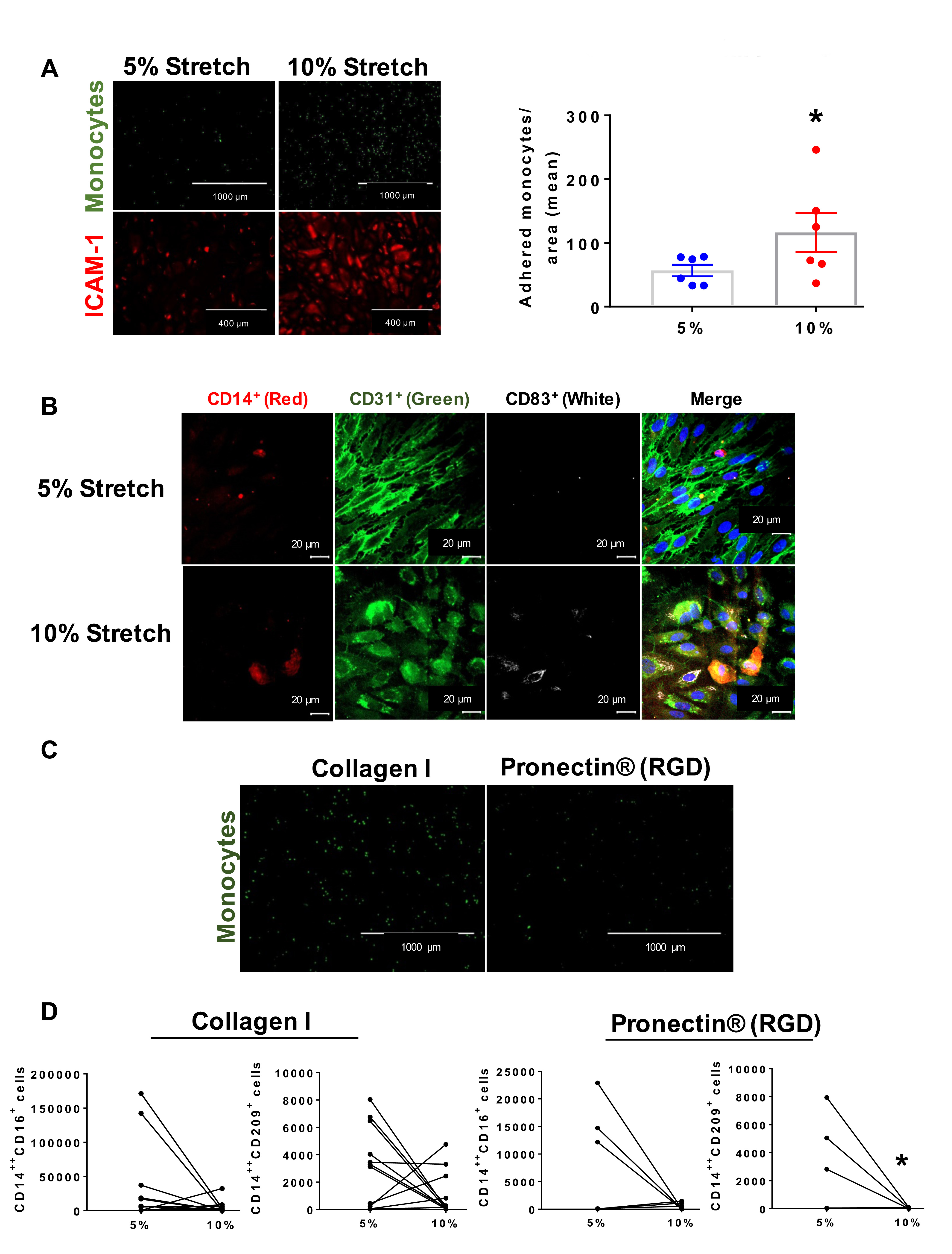

Supplement: Supplementary Fig 5 [file cvy112_supplementary_fig_5.jpeg]

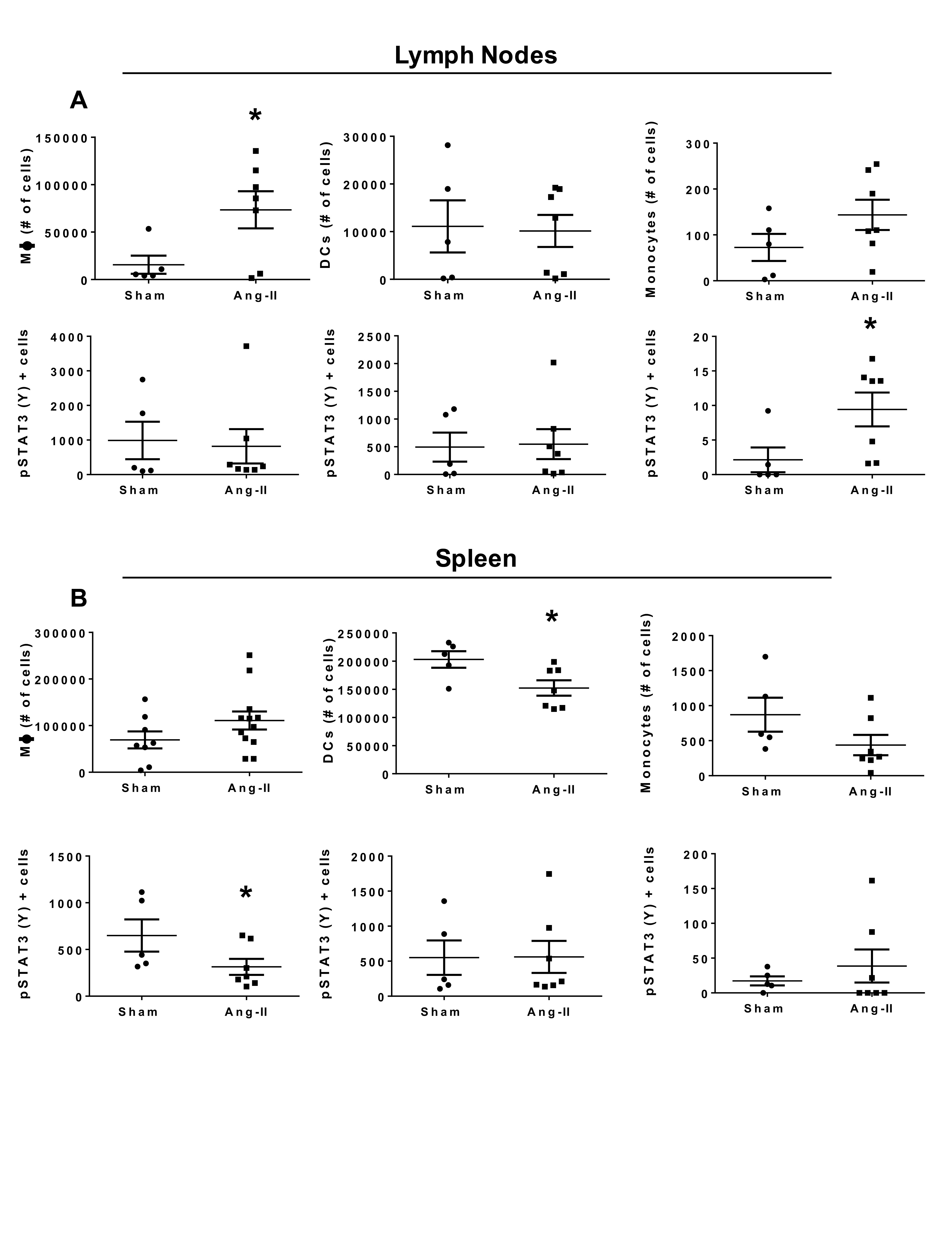

Supplement: Supplementary Fig 6 [file cvy112_supplementary_fig_6.jpeg]

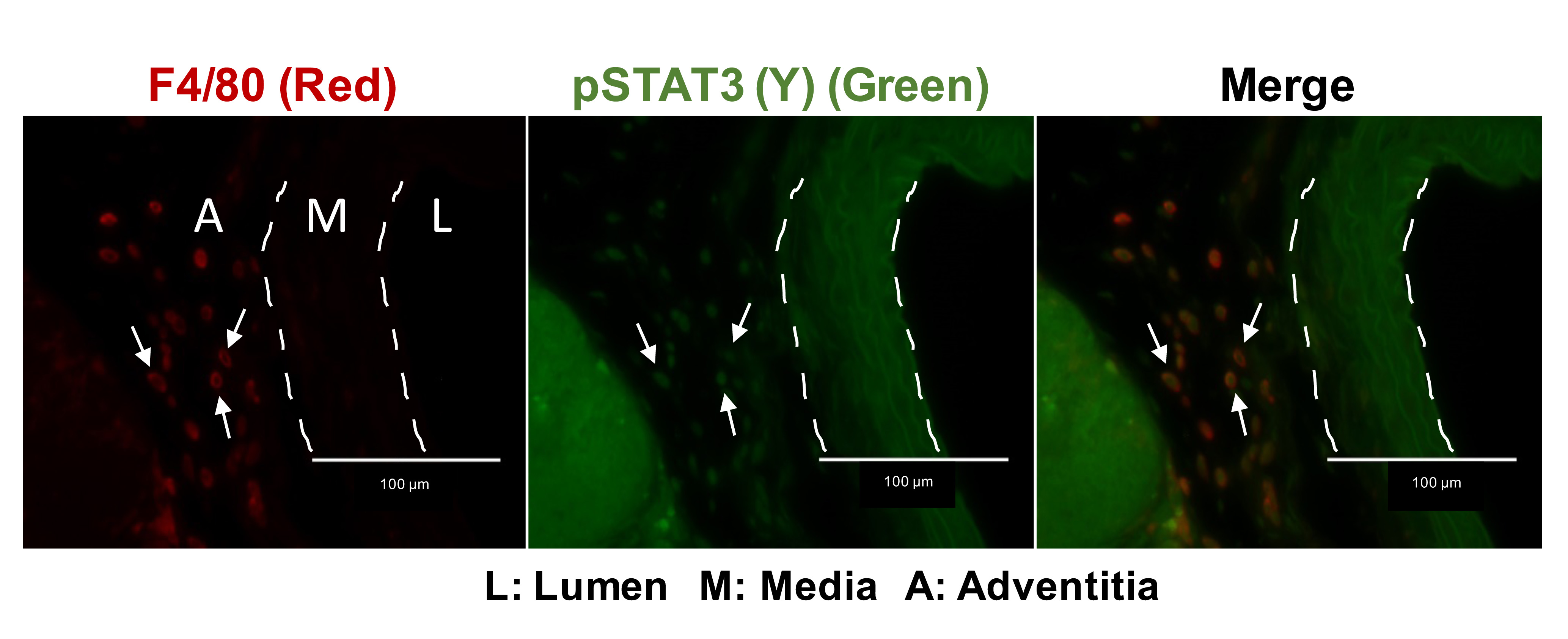

Supplement: Supplementary Fig 7 [file cvy112_supplementary_fig_7.jpeg]
