# Supplementary material for: Hypertension and increased endothelial mechanical stretch promote monocyte differentiation and activation: roles of STAT3, interleukin 6 and hydrogen peroxide
Source: Cardiovasc Res. 2018 May 23;114(11):1547–63. doi: 10.1093/cvr/cvy112 (PMC6106108; doi:10.1093/cvr/cvy112)
Supplement: Supplementary Data [file cvy112_supplementary_data.docx]

**SUPPLEMENT**

**Hypertension and increased endothelial mechanical stretch promote monocyte differentiation and activation: Roles of STAT3, interleukin 6 and hydrogen peroxide**

Roxana Loperena^1^, Justin P. Van Beusecum^2^, Hana A. Itani^2^, Noah Engel^3^, Fanny Laroumanie^2^, Liang Xiao^2^, Fernando Elijovich^2^, Cheryl L. Laffer^2^, Juan S. Gnecco^5^, Jonathan Noonan^6^, Pasquale Maffia ^4, 6, 7^, Barbara Jasiewicz-Honkisz^8^, Marta Czesnikiewicz-Guzik^4^, Tomasz Mikolajczyk^8^, Tomasz Sliwa^8^, Sergey Dikalov^2^, Cornelia Weyand,^9^ Tomasz J. Guzik^4^, and David G. Harrison^1,2^

^1^Department of Molecular Physiology and Biophysics, Vanderbilt University, Nashville, TN

^2^Division of Clinical Pharmacology, Department of Medicine, Vanderbilt University Medical Center, Nashville, TN

^3^Department of Biological Sciences, Vanderbilt University, Nashville, TN

^4^Institute of Cardiovascular and Medical Sciences, University of Glasgow, Glasgow, UK

^5^Department of Pathology, Microbiology and Immunology, Vanderbilt University, Nashville, TN

^6^Institute of Infection, Immunity & Inflammation, University of Glasgow, Glasgow, UK

^7^Department of Pharmacy, University of Naples Federico II, Naples, Italy

^8^Department of Internal Medicine and Department of Immunology Jagiellonian University School of Medicine, Cracow Poland,

^9^Division of Immunology and Rheumatology, Department of Medicine, Stanford University School of Medicine, Palo Alto, CA

**Address for correspondence:**

David G. Harrison, MD
2200 Pierce Avenue

Room 536 Robinson Research Building
Vanderbilt University
Nashville, TN 37232-6602

Telephone 615-322-3304/ Fax 615-875-3297
e-mail [david.g.harrison@vanderbilt.edu](mailto:david.g.harrison@vanderbilt.edu)

**Supplemental Figure 1: Intermediate levels of endothelial stretch and effects on monocyte phenotype. A.** Human CD14^+^ monocytes were isolated from PBMCs of normal participants and cultured with HAECs undergoing 5% (n = 10), 6% (n = 4), 8% (n = 3) and 10% (n = 10) cyclical stretch for 48 hours. Mean data showing the percent number of intermediate monocytes in response to each stretch percentile. **B.** Percent of live monocytes in paired experiments after 48-hour exposure to endothelial cells undergoing either 5% or 10% stretch. Comparisons were made using one-way ANOVA with Student Newman Keuls post-hoc test in panel A and one-tailed paired t-tests for panel B (****p*<0.001, *****p*<0.0001).

**Supplemental Figure 2: Hypertensive mechanical stretch in human endothelial cells effects on monocyte gene expression.** Human CD14^+^ monocytes were isolated from PBMCs of normal human volunteers and cultured with HAECs exposed to 5% or 10% stretch for 48 hours. Relative monocyte mRNA expression of TGFβ-1 (n=8), MMP8 (5%, n=3; 10%, n=4), CCL2 (n=5), IL-18 (n=5) and CD168 (n = 8) in monocytes. Statistical differences were determined using one-tailed unpaired t-tests (***p*<0.01).

**Supplemental Figure 3: STAT3 and STAT1 expression in other monocyte subpopulations when culture with endothelial cells undergoing mechanical stretch.** Human CD14^+^ monocytes were isolated from PBMCs of normal human volunteers and cultured with HAECs exposed to 5% or 10% stretch for 48 hours. **A**. Individual values for number of cells within the classical monocyte population expressing p-STAT3 (Y), p-STAT3 (S), and p-STAT1 for each subject are indicated by connected lines (n= 9). **B**. Values for total number of cells within the CD14^low^CD16^++^ non-classical population expressing p-STAT3 (Y), p-STAT3 (S), and p-STAT1 for each participant (n = 8). **C.** Proteins from monocyte cell lysates (20 µg) in cultures with HAECs exposed to 5% or 10% stretch after 48 hours were immunoprecipitated with total STAT3 and subjected to Western Blot detection of total STAT1 protein. Representative of two independent experiments are shown (n=8). Statistical differences were determined using one-tailed paired t-tests (**p*<0.05).

**Supplemental Figure 4: Interleukin 6 is released by endothelial cells undergoing hypertensive mechanical stretch.** Human aortic endothelial cells were grown to confluency and exposed to either 5% or 10% stretch for 48 hours. Conditioned media from HAECs exposed to various levels of stretch was used to detect IL-6 by ELISA and normalized according to a concentration curve. Mean values for IL-6 are shown as pictogram per milliliter (n= 9). Statistical difference was determined using a one-tailed unaired t-test. (**p*<0.05).

**Supplemental Figure 5: Human monocytes require endothelial cell contact for their differentiation and activation during hypertensive mechanical stretch. A.** Human monocytes from normal volunteers were isolated from buffy coats and labeled with 12µM of CellTracker™ Green CMFDA dye for 30 mins at 37°C in medium and added to HAEC cultures exposed to either 5% or 10% stretch for 24 hours. Plates were washed with 1X PBS and fixed with 4% PFA. Adhered monocytes were counted. Immunofluorescent microscopic images show monocytes adhered to the endothelial cells (green) and expression of ICAM-1 on the endothelial cell monolayer (red). Mean data showing the number of adhered monocytes per three random fields are illustrated (n=6). **B**. Human monocytes from normal volunteers were isolated from buffy coats and added to HAEC cultures exposed to either 5% or 10% stretch for 24 hours. Plates were washed with 1X PBS and fixed with 4% PFA. Confocal microscopy with Z stacking was used to visualize CD31^+^ (green), CD14^+^ (red) and CD83^+^ (white) in different surfaces including the subendothelial space. Cells were counterstained with DAPI (n=4). **C.** Human CD14^+^ monocytes labeled with 12 µM of CellTracker™ Green CMFDA dye were placed on either collagen I or Pronectin® (RGD) coated plates without the presence of endothelial cells and were exposed to 5% or 10% cyclical stretch for 24 hours. Immunofluorescent microscopic images show monocytes that adhered to the collagen I and Pronectin® coated membranes after exposure to hypertensive mechanical stretch (n= 4). **D.** Human CD14^+^ monocytes were cultured alone on collagen I (n=11) or Pronectin® (n=6) coated plates and stretched to either 5% or 10% for 48 hours. Individual values are shown for each participant of the number of cells expressing CD14^++^CD16^+^ and CD14^++^CD209^+^ cells in each experimental condition. Statistical differences were determined using one-tailed unpaired or paired t-tests (**p*<0.05).

**Supplemental Figure 6: Angiotensin II-induced hypertension in wildtype C57 mice and the STAT3 phosphorylation in the immune cells from lymph nodes and spleen.** C57 Bl/6 wildtype mice were infused with Ang II (490 ng/kg/min) or sham for 6 days. **A.** Mean values of absolute numbers of indicated cell types per periaortic lymph nodes. Mean values of p-STAT3 (Y) expression within the macrophage (Mφ), DC and monocyte populations per periaortic lymph nodes. **B.** Mean values of absolute numbers of indicated cell types per spleen. Mean values of p-STAT3 (Y) expression within the macrophage, DC and monocyte populations per spleen. Statistical differences were determined using one-tailed unpaired t-tests (**p*<0.05).

**Supplemental Figure 7: Monocytes and macrophages from angiotensin-II hypertensive wildtype mice localize to the perivascular fat in the aorta and express STAT3.** C57BL/6 mice were infused with Ang II (490 ng/kg/min) or sham for 14 days. Perfusion-fixed sections of the thoracic aortas were sectioned (6 mm) and immunofluorescence was performed to stain for F4/80 (red), pSTAT3 (Y705) (green) and counterstained with DAPI. Co-localization of F4/80 and pSTAT3 (Y) is shown.

**Supplemental Table I: Power achieved for each experimental condition**

* For intermediate monocytes; ^τ^ for pSTAT3 + dendritic cells in aorta; ᵵ for pSTAT3 (Y) MFI from normotensive human intermediate vs classical monocytes.
